# Protein overabundance is driven by growth robustness

**DOI:** 10.1126/sciadv.adz9623

**Published:** 2026-03-20

**Authors:** H. James Choi, Teresa W. Lo, Kevin J. Cutler, Dean Huang, William Ryan Will, Paul A. Wiggins

**Affiliations:** ^1^ Department of Physics, University of Washington, Seattle, WA 98195, USA.; ^2^ Department of Laboratory Medicine and Pathology, University of Washington, Seattle, WA 98195, USA.; ^3^ Department of Microbiology, University of Washington, Seattle, WA 98195, USA.; ^4^ Department of Bioengineering, University of Washington, Seattle, WA 98195, USA.

## Abstract

Protein expression levels optimize cell fitness: Too low an expression level of essential proteins will slow growth by compromising essential processes, whereas overexpression slows growth by increasing the metabolic load. This trade-off naïvely predicts that cells maximize their fitness by sufficiency, expressing just enough of each essential protein for function. We test this prediction in the naturally competent bacterium *Acinetobacter baylyi* by characterizing the proliferation dynamics of essential-gene knockouts at a single-cell scale (by imaging) as well as at a genome-wide scale. In these experiments, cells proliferate for multiple generations as target protein levels are diluted from their endogenous levels. This approach facilitates a proteome-scale analysis of the fitness landscape with respect to protein abundance. We find that most essential proteins are subject to a threshold-like fitness landscape: Growth is independent of protein abundance above a critical threshold and arrests below that threshold. We have recently analyzed the implications of this landscape for growth robustness. Confirming signature predictions of this model, we find that (i) roughly 70% of essential proteins are overabundant, (ii) overabundance increases as the expression level decreases, and (iii) the lowest abundance proteins are in vast excess (>10×) of what is required for growth in the typical cell. These results reveal that robustness plays a fundamental role in determining the expression levels of essential genes and that overabundance is a key mechanism for ensuring robust growth.

## Teaser

Fitness optimization drives vast overabundance in low-expression proteins.

## INTRODUCTION

Understanding the rationale for protein expression levels is a fundamental question in biology with broad implications for understanding cellular function (*1*). Measured expression levels appear to be paradoxically both optimal and overabundant. For instance, repeated investigations support the idea that gene expression levels optimize cell fitness (*2*, *3*). Given that the overall metabolic cost of protein expression is large (*4*, *5*), fitness optimization would seem to imply that protein levels should satisfy a Goldilocks condition: Expression levels should be just high enough to achieve the required protein activity (*6*, *7*). However, a range of approaches suggest that many essential genes are expressed in vast excess of the levels required for function (*7*–*9*). How can expression levels be at once optimal with respect to fitness as well as in excess of what is required for function?

The cell faces a complex regulatory challenge: Even in a bacterium, there are between 400 and 600 essential proteins, each of which is required for growth (*10*). How does the cell ensure the robust expression of each essential factor? We recently argued that the stochasticity of gene expression processes fundamentally shapes the principles of central dogma regulation, including the optimality of protein overabundance (*11*). Specifically, we proposed a quantitative model, the robustness-load trade-off (RLTO) model, which makes a parameter-free prediction of protein overabundance as a function of gene transcription level (*11*). The optimality of overabundance can be understood as the result of a highly asymmetric fitness landscape: The fitness cost of essential protein underabundance, which causes the arrest of essential processes, is far greater than the fitness cost of essential protein overabundance, which leads to slow growth by increasing the metabolic load. However, critical model assumptions and predictions remain untested, which is the motivation for the current study. Here, we will quantitatively measure the fitness landscape with respect to protein abundance and determine the level of overabundance for all essential proteins in the bacterium *Acinetobacter baylyi*.

## RESULTS

### Natural competence facilitates knockout depletion

**Fig. 1.**
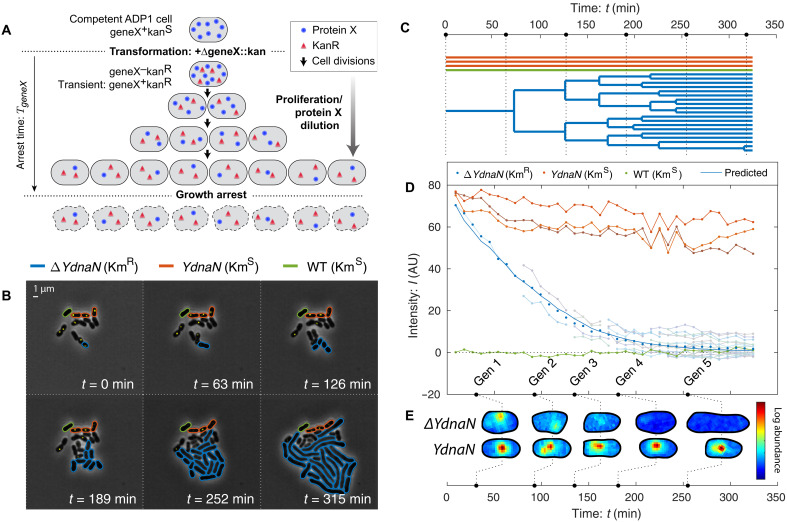
Knockout-depletion experiments. (**A**) Experimental schematic. Competent ADP1 cells are transformed with Δ*geneX::kan*. Untransformed cells arrest immediately on selective media. Transformed cells proliferate but cease protein X expression (blue circles) while expressing Kan (red triangles). Existing protein X abundance is diluted as cells proliferate. For essential genes, cell growth continues until protein levels are diluted to the threshold level required for growth, after which growth arrests. (**B** and **C**) Visualization of knockout depletion. The fluorescent fusion *YPet-dnaN* to essential gene *dnaN* is knocked out at *t* = 0. Cell proliferation is visualized using phase-contrast microscopy, while protein abundance is measured by fluorescence microscopy (yellow). Transformed cells (Δ*YdnaN*, blue) have a Km*^R^* allele and can proliferate over several generations before arrest; however, untransformed cells (*YdnaN*, orange) and wild-type cells (WT, green) were both kanamycin-sensitive and therefore arrested immediately (see section 2A2). (C) Lineage tree. Black dotted lines represent time points shown in (B). (**D**) Target protein is diluted by proliferation. The protein concentration is measured by integrated fluorescence. Arrested *YdnaN* cells maintain protein abundance, whereas proliferating transformed cells (Δ*YdnaN*, blue) show growth-induced protein depletion. The protein concentration over all transformed progeny (blue points) is consistent with the dilution-model prediction (solid blue). AU, arbitrary units. (**E**) The protein function is robust to dilution. Representative single-cell images of transformed (Δ*YdnaN*) and untransformed (*YdnaN*) cells are shown for successive time points. The YPet-DnaN fusion shows punctate localization, consistent with function, even as protein abundance is depleted. No puncta are observed in the last generation, and the cells form filaments, consistent with the replication arrest phenotype.

To characterize the fitness landscape for essential gene expression, we must deplete the levels of essential proteins. Both degron- and CRISPR interference (CRISPRi)–based approaches have been applied; however, these approaches require careful characterization of protein levels (*8*, *12*–*15*) and could introduce substantial cell-to-cell variation at the single-cell scale (*16*), on top of the endogenous gene expression noise, which further obscures the underlying fitness landscape. To circumvent these difficulties, we will use an alternative approach: knockout depletion in the naturally competent bacterium *A. baylyi* ADP1 (*17*, *18*). *A. baylyi* natively takes up and integrates extracellular DNA from its environment without artificial treatment (*19*, *20*). In the knockout-depletion approach, these cells are transformed with *geneX::kan* knockout cassettes targeting essential gene X, carrying a kanamycin resistance allele Km*^R^* (see Fig. 1A). Cells that are not transformed arrest immediately on selective media. The crux of the approach is that transformants remain transiently *geneX*^+^ because of the presence of already synthesized target protein X, even after the transcription of the target *geneX* stops. Growth can continue, diluting protein X abundance, as long as this residual abundance remains sufficient for function. The success of the knockout-depletion approach is dependent on the extremely high transformation efficiency of *A. baylyi*.

### Target proteins are depleted by dilution

A key untested assumption in the experimental design of the knockout-depletion approach is that target protein translation stops after transformation and that the protein abundance is depleted by dilution. The model predicts that the relative protein concentration is

$$C\left(t\right)/C_{0}=V_{0}/V\left(t\right)$$ (1)

where *C*_0_ and *V*_0_ are the concentration and volume of the progenitor cell at deletion, respectively, and *V*(*t*) is the total volume of the progeny. To test the predicted protein depletion hypothesis, we designed a knockout-depletion experiment to target a protein we had previously studied that can be visualized using fluorescent fusion and whose localization is activity dependent: the essential replication gene *dnaN*, whose gene product is the β sliding clamp (*21*–*23*). We constructed a N-terminal fluorescent fusion to *dnaN* using YPet in *A. baylyi* at the endogenous locus. The resulting mutant (YdnaN) had no measurable growth defect under our experimental conditions. We then knocked out the *YPet-dnaN* fusion, yielding Δ*dnaN*, and characterized the protein levels by quantifying YPet-DnaN abundance by fluorescence.

The experimental design of knockout-depletion assays (Fig. 1A) can fail by a number of distinct mechanisms: (i) Transient growth of kanamycin-sensitive cells on selective media could be misinterpreted as the transient growth of transformants. Three lines of evidence argue against this possibility. No transient growth is observed in cells that are transformed with fragments without the *kan* gene. Furthermore, progeny of cells transformed with *kan*-carrying knockout cassettes exhibits consistent phenotypes (e.g., the cell lysis phenotype for Δ*murA*). Last, Kan expression appears to be overabundant itself, as discussed in section S1A.

(ii) Competent cells could exhibit protein expression in vast excess of log-phase cells. To rule out this possibility, we measured YPet-DnaN abundance in competent and wild-type cells. We observed that competent cells in outgrowth have comparable protein abundance to log-phase cells (see section S1B). (iii) Another important consideration is the rapidity of transformation and the recombination processes. If the DNA target sequence is not rapidly degraded and expression continues, transient growth could be the consequence of this continuing expression. To rule out this possibility, we determined the dilution rate in four microcolonies, and in each case, it was consistent with the rate cell elongation. (We note that two roughly canceling competing effects limit the precision of this analysis: photobleaching and the fluorescence from neighboring wild-type cells. See section S1C.) In additional support to experimental design, dilution is observed immediately on the first cell division, suggesting that the target gene is either rapidly degraded or at least cannot be transcribed (see Fig. 1D).

(iv) Another complicating scenario is the possibility that the protein is not partitioned equally between daughter cells. Contrary to this possibility, we have previously studied the partitioning of nearly all localized proteins in *Escherichia coli* and quantified protein partitioning between daughter cells. Only proteins localized in polar puncta were asymmetrically partitioned (*24*). Consistent with these measurements in *E. coli*, *A. baylyi* partitions YPet-DnaN nearly equally between daughters early in proliferation. Late in proliferation, a technical limitation, fluorescence from neighboring wild-type cells, limits the ability to quantitatively measure partitioning; however, growth arrest in progeny after the initial cell division appears synchronous, consistent with equipartition of mother-cell protein (see Fig. 1D). Observations (i) to (iv) confirm that proteins are diluted, consistent with the experimental design shown schematically in Fig. 1A.

### Replication persists during DnaN depletion

A key subhypothesis of the overabundance model for transient growth is that target protein function continues as the target protein abundance is depleted. An alternative hypothesis for the transient growth of the Δ*dnaN* strain is a high initial chromosomal copy number that is partitioned between daughter cells, even after the replication process itself arrests because of target protein depletion (*4*, *25*). The imaging-based knockout-depletion experiment tests this hypothesis as well. The localization of DnaN is dependent on activity: During ongoing replication, DnaN is localized in puncta corresponding to replisomes, whereas in the absence of active replication, DnaN has diffuse localization (*21*–*23*, *26*, *27*). During the knockout-depletion experiment, we observed that YPet-DnaN puncta persist as the targeted fusion was depleted (Fig. 1, D and E), consistent with replication activity after dilution. Only after the YPet-DnaN puncta disappear do the cells begin to adopt the Δ*dnaN* phenotype: cell filamentation (Fig. 1, B and E). We therefore conclude that function (replication) is robust to substantial target protein (DnaN) dilution.

### Other essential knockouts undergo transient growth

**Fig. 2.**
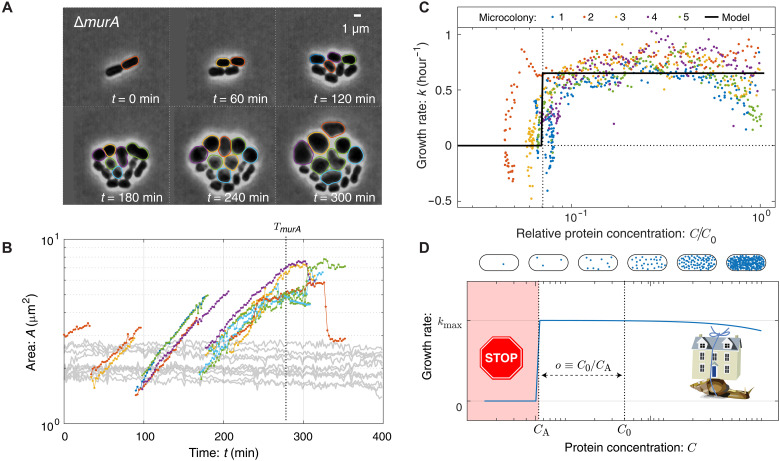
Fitness landscape. (**A**) Visualization of growth in a *murA* knockout. Essential gene *murA* is knocked out at *t* = 0, and cell proliferation is visualized by phase-contrast microscopy. Cell proliferation continues for multiple generations after deletion. (**B**) Quantitative analysis of cell proliferation with a single-cell resolution. Cell area (log scale) as a function of time for the *murA* deletion (colored lines) and representative untransformed cells (light gray). Each color represents the progeny of the initial mutant. The log slope represents the single-cell growth rate. The vertical dotted line represents the arrest time at which transformant growth slows to growth arrest. Untransformed cells are arrested throughout the experiment, given that the cells are Km^S^. (**C**) Growth rate as a function of protein depletion for Δ*murA*. Both the relative protein abundance (Eq. 1) and single-cell growth rate (Eq. 2) are inferred from the observed cell volume. The growth rate is observed to obey a threshold-like dependence on protein abundance, transitioning between wild-type growth to arrest at the vertical dotted line. We define the critical dilution as $o\equiv C_{0}/C_{A}$, where $C_{A}$ is the protein concentration at arrest. Cell-to-cell variation is observed in the arrest of four microcolonies that originate from distinct progenitor cells. The model represents a threshold-like model fit to the growth rate data. (**D**) The fitness landscape is threshold-like. Motivated by single-cell growth data, cell fitness is modeled using the RLTO model. In the model, there is a metabolic cost of protein expression, which favors low expression; however, growth arrests for protein concentration *C* smaller than the threshold level $C_{A}$ (red). The relative metabolic cost of overabundance is small relative to the cost of growth arrest because of the large number of proteins synthesized, resulting in a highly asymmetric fitness landscape (*11*).

To understand the generic consequences of essential protein depletion, we used the imaging-based knockout-depletion experiments to explore essential genes with a range of functions. We initially targeted four essential genes: the replication initiation regulator gene *dnaA* (fig. S4 and movies S5 and S6), the β-clamp gene *dnaN* (fig. S5 and movies S7 and S8), the cell-wall-synthesis gene *murA* (fig. S7 and movies S9 and S10), and septation-related gene *ftsN* (fig. S6 and movies S11 and S12), as well as a nonessential insertion sequence element with no phenotype as a negative control (movie S3 and S4). In each case, transformants continued to proliferate through multiple cell-cycle durations (*17*) and are therefore consistent with the essential protein overabundance hypothesis. However, in (*17*), we were unable to perform a quantitative single-cell analysis of these time-lapse experiments because existing segmentation packages failed to segment the observed morphologies (*28*). We therefore developed the Omnipose package, which facilitated quantitative analysis of the growth dynamics with a single-cell resolution (see Figs. 1B and 2A) (*28*).

### The fitness landscape is highly asymmetric

A key input to the RLTO model is the fitness landscape (growth rate) as a function of protein abundance. Omnipose segmentation facilitates the measurement of single-cell growth rates from the time-lapse imaging experiments. We focus first on the single-cell areal growth rate

$$k\left(t\right)=\frac{d}{\mathrm{dt}}\text{ln}\;A\left(t\right)$$ (2)

where *A*(*t*) is the area of the cell at time *t*. This areal growth rate is more convenient than a cell length–based rate because we avoid the necessity of defining cell length for unusual cell morphologies like those observed in the Δ*murA* mutant. Figure 2B shows representative knockout-depletion dynamics of cell area for the essential-gene target *murA*. The log slope remains constant for multiple generations, consistent with a constant growth rate, even as the gene targeted is depleted over multiple cell cycles. By combining the dilution model (Eq. 1) and the growth rate (Eq. 2), a single knockout-depletion measurement determines the growth rate for a range of protein abundances between wild-type abundance and those realized at growth arrest. This fitness landscape is shown for the MurA protein in Fig. 2C. For all four mutants, the areal growth rate is roughly constant for multiple generations before undergoing a rapid transition to growth arrest (see section S1). We emphasize that the predictions of the RLTO model do not depend on a rigorous step-like transition of the growth rate to zero but rather a rapid decrease in growth rate under a threshold protein abundance as observed for each target (*11*).

### Protein overabundance

We will define the overabundance as the ratio of the initial protein concentration (*C*_0_) to the concentration at cell arrest (*C*_A_)

$$o\equiv C_{0}/C_{A}$$ (3)

as shown in Fig. 2D (see Materials and Methods). Note that in principle, both *C*_0_ and *C*_A_ could be determined independently; however, what is measured in the knockout-depletion experiment is the ratio *o*, defined as the overabundance, only. The measured overabundance for the four mutants imaged by microscopy is summarized in Table 1 using three distinct metrics for growth. We conclude that for each gene, with the exception of *dnaA*, rapid growth continues after the knockout because of the vast overabundance of the target protein.

**Table 1.** Measured overabundance for sequencing-based versus imaging-based approaches. The overabundance was determined by both sequencing- and imaging-based approaches. Each of the three different overabundance measurements is inferred from the arrest of a distinct cellular process: The sequencing-based measurement is determined from dynamics of genomic copy number, which depends on replication. For the imaging-based approach, we show two measurements on the basis of different metrics for arrest: The first is based on the arrest of cell elongation, as defined by Eq. 3, and the second is based on the arrest of the septation process, as visualized by microscopy. *N*_c_ and *N*_p_ are the number of cells analyzed and the number of progenitor cells analyzed in the imaging-based experiments, respectively. For sequencing-based measures, errors are estimated from the analysis of replicate experiments. For imaging-based errors, errors are estimated from the variance between arrest times for distinct progenitor cells (see table S1 for a more detailed description of analysis).

Gene	Annotated gene function	Transcription: μ_m_ (mRNA molecules/cell cycle)	Log overabundance			
			TFNseq replication	Imaging-based elongation septation		
			log_10_*o*	log_10_*o*	log_10_*o*	(*N*_c_,*N*_p_)
*dnaA*	Regulation of replication initiation	30	0.02 ± 0.02	0.7 ± 0.1	0.0 ± 0.2	(4,4)
*dnaN*	Replication β sliding clamp	49	1.5 ± 0.1	2.0 ± 3.0	1.4 ± 0.1	(134,8)
*ftsN*	Essential cell division/septation protein	20	2.6 ± 0.1	1.8 ± 0.2	0.6 ± 0.2	(19,5)
*murA*	Cell wall precursor synthesis	26	0.7 ± 0.5	1.1 ± 0.1	0.9 ± 0.2	(16,4)

### The RLTO model predicts protein overabundance

The RLTO model explicitly analyzes the trade-off between growth robustness to noise and metabolic load and predicts the optimal central-dogma regulatory principles (*11*). Critically, the model incorporates the observed threshold-like dependence of growth rate on protein abundance (Fig. 2, C and D). The model quantitatively predicts protein overabundance with a signature feature: High-expression genes have low protein overabundance (o≈1) because of the high metabolic cost of increasing expression and low inherent noise of high-expression genes; however, low-expression genes have high overabundance (o≫1) because of the low metabolic cost of increasing expression and the high inherent noise of low-expression genes (see section S5 for a more detailed description of the model).

### TFNseq determines overabundances genome-wide

**Fig. 3.**
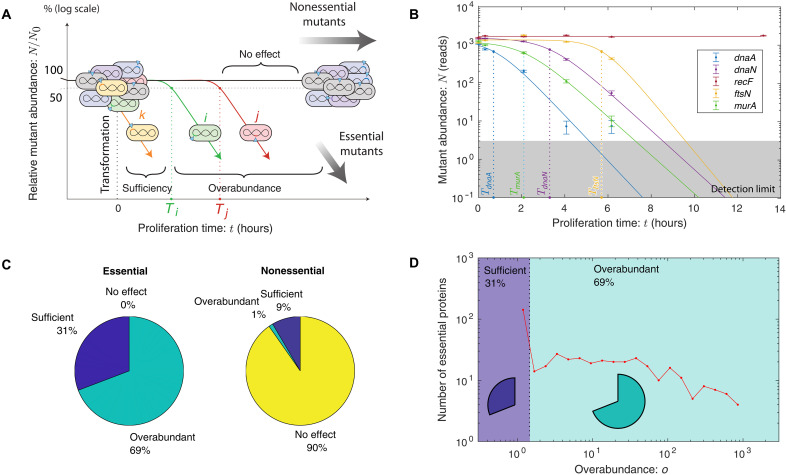
Proteome-wide analysis of protein overabundance. (**A**) TFNseq schematic. A polyclonal library of knockout mutants is generated by the transformation of ADP1 with DNA mutagenized by transposon insertions. The library is proliferated on selective media, and sequential fractions are collected. The relative-abundance trajectories of mutants are determined by mapping transposon insertion sites by sequencing. The arrest time parameter $T_{i}$ corresponds to the time at which the growth rate changes from wild-type to slow growth for mutant *i*. The arrest times are represented as dotted lines, corresponding to the time at which the relative abundance falls to 50% its initial value (see section S2B). (**B**) TFNseq-trajectory analyses for five mutant strains. Each mutant trajectory is well fit by one of the three trajectory models. As expected, the no-effect model is selected for the nonessential gene *recF*. For the other four essential genes, the overabundance model is selected. The dotted line represents the arrest time for each mutant. (**C**) Proteome-wide analysis of proliferation dynamics. For genes classified as essential, 31% were best fit by the sufficiency-dynamics model, while 69% were best fit by the overabundance trajectory model. For genes classified as nonessential, 90% were best fit by the no-effect model, while 10% showed a detectable reduction in growth rate. (**D**) Overabundance varies by orders of magnitude between essential proteins. The protein overabundance is inferred from the arrest time using Eq. 4. Sufficient expression genes have overabundance *o* = 1, while overabundant genes vary from *o* > 1 to very large overabundance (*o* > 100).

To test the signature expression-dependent overabundance prediction of the RLTO model, we now transition to a genomic-scale analysis. The Manoil lab developed a transformation transposon insertion mutant sequencing (TFNseq) approach to knockout-depletion experiments for targeting all genes simultaneously in *A. baylyi* (*18*). In short, a genomic library was prepared and mutagenized using a transposon carrying the Km*^R^* allele. The resulting DNA was then transformed into *A. baylyi*. The transformants were propagated on selective liquid media, and fractions were collected every 2 hours from which genomic DNA was extracted. The transposons were then mapped using TFNseq to generate the relative abundance trajectory for each mutant (see Fig. 3, A and B) (*18*). We then analyzed each mutant trajectory statistically using three competing growth models: no effect, sufficiency, and overabundance using two successive null-hypothesis tests (see section S3). For each mutant *i* described by the overabundance model, the TFNseq experiment measures a growth arrest time *T_i_* and the corresponding target protein overabundance

$$o_{i}=\text{exp}\left(k_{0}T_{i}\right)$$ (4)

where *k*_0_ is the wild-type growth rate. Two replicate experiments lead to comparable overabundance estimates (see section S3).

To test the consistency of this TFNseq approach with imaging-based knockout-depletion measurements, we focused first on the analysis of the mutants *dnaA*, *dnaN*, *ftsN*, and *murA*. As shown in Fig. 3B, the trajectories for *dnaA*, *murA*, *ftsN*, and *dnaN* show an unambiguous step-like change in growth dynamics: The no-effect trajectory model (null hypothesis) is rejected with *P* values that are below machine precision, and the sufficiency trajectory model is also rejected with *P* < 10^−4^ for all genes. In Table 1, we compare protein overabundances determined by imaging- and sequencing-based approaches. These numbers are qualitatively consistent. For instance, the single-cell analysis of the *dnaA* mutant shows a nearly immediate phenotype by imaging (i.e., cell filamentation) (see section S1E). Likewise, the TFNseq approach finds an overabundance of 1.0, meaning that protein expression is sufficient. On the other hand, all three of the other mutants (*murA*, *ftsN*, and *dnaN*) are found to have very large overabundances and are roughly comparable. Last, a representative nonessential gene (e.g., *recF*) shows no effect. These results support the use of the TFNseq approach to analyze protein overabundance genome-wide.

### Many essential proteins have vast overabundance

To determine the protein overabundance genome-wide, we analyzed the knockout-depletion trajectories for all genes in *A. baylyi* (see Fig. 3, B to D). Our analysis showed that the vast majority (90%) of genes annotated as nonessential were classified as having no effect and 10% of nonessential genes had measurable growth defects. (Replicate experiments were used to estimate the error in the protein overabundance. See fig. S13.) The most severe growth defect in nonessential annotated genes were observed for the genes *gshA* and *rplI*.

For the essential gene analysis, we defined essential genes conservatively, demanding that they be classified essential by both TFNseq (*18*) and single-gene deletion experiments (432 genes) (*29*). All essential mutants were observed to have growth defects, as anticipated; however, only 31% of essential proteins were classified as sufficient, corresponding to an immediate change in growth rate, and are assigned overabundance *o* = 1 corresponding to the natural protein abundance being close to the threshold value. Notable genes in this category include ribosomal proteins RpsQ and RpsE, ribonucleotide reductase subunits NrdA and NrdB, and adenosine 5′-triphosphate synthase subunits AtpA and AtpD. However, as predicted by the RLTO model, most essential proteins (69%) were classified as overabundant, meaning that they required substantial dilution before a growth rate change was detected. Figure 3D shows a histogram of essential gene overabundances.

### Low-transcription genes are highly overabundant

To understand the overall significance of overabundance in a typical biological process, we determined the median essential protein overabundance: sevenfold. To understand the significance of overabundance from the perspective of the metabolic load, we also determine the mean protein overabundance, weighted by the expression level: 1.6-fold. These two superficially conflicting statistics emphasize a key predicted regulatory principle: Overabundance is high for low-abundance proteins; however, it is close to unity for the high-abundance proteins, which constitute the dominant contribution to the metabolic load.

**Fig. 4.**
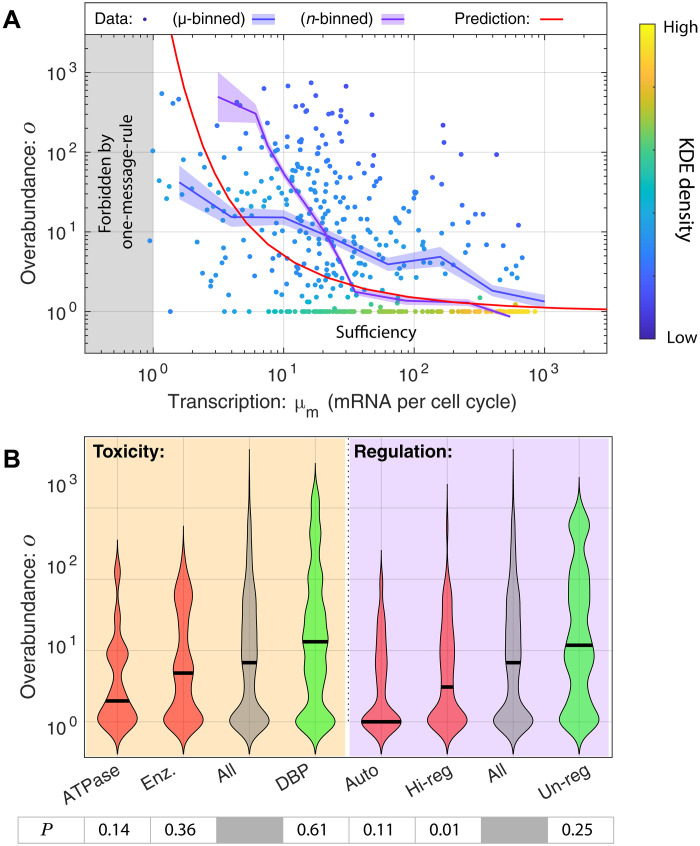
Protein overabundance depends on expression, regulation, and protein function. (**A**) Overabundance is large for low-expression essential proteins. The measured transcription-overabundance pairs are shown for all essential genes. [The density of data points was estimated with a KDE (kernel density estimation), which highlights the pileup of genes in the bottom right-hand corner of the plot.] To analyze the trend, these data were binned using two complementary approaches (blue and purple) (see section S3C.) The RLTO model (red) predicts that overabundance grows rapidly as the transcription level is reduced. The RLTO model (red) qualitatively captures the trend of the data; however, it appears to underestimate the measured overabundance for intermediate expression genes. The RLTO model predicts that essential genes cannot be expressed below the level of one mRNA per cell cycle (see Discussion) (*11*). Points lying on the *o* = 1 line are classified as sufficient. (**B**) Toxicity and regulation are determinants of overabundance. We compared the overabundance measurements for six essential gene subgroups to determine whether toxicity and regulation could affect overabundance. Red groups were predicted to decrease overabundance, while green groups were expected to increase it. The *P* values for the consistency of each distribution with the all-gene group are shown below each category and are generated using the Kolmogorov-Smirnov test (*46*). As hypothesized, the data are consistent with both toxicity and regulation decreasing overabundance.

To explicitly test the predicted relation between protein expression and overabundance, we quantified transcription by measuring the relative abundance of mRNA messages by RNA sequencing for exponentially growing *A. baylyi* cells (see section S4). We estimated transcription in mRNA transcripts per gene per cell cycle (message number) for each essential gene (see section S4B). Figure 4A compares transcription and overabundance for all essential genes with the prediction of the RLTO model. To quantitatively capture the trend, we applied two different binning approaches to compare the data cloud to the RLTO model predictions. As predicted, the data show a clear trend of decreasing overabundance with increasing message number. With very few exceptions, high-expression genes have extremely low overabundance. At the other extreme, low-expression genes typically have large to very large overabundance as shown by the sharp up-turn of the purple curve as the transcription level approaches the one-message-rule threshold, a lower threshold on essential-gene transcription that we recently proposed (*11*).

### Limitations of knockout-depletion experiments

In spite of the success of the RLTO model in predicting the genomic-scale overabundance trend, there are many significant outliers from this prediction. In considering their significance, it is important to emphasize the flaws both with the knockout-depletion experiments and the RLTO model. With respect to the experiments, the mechanism of growth arrest plays an important role in determining which growth metric most accurately determines the arrest time. Consider the three overabundances (quantified from arrest time) measured for the septation-related essential gene *ftsN* in Table 1. Because of the absence of strict cell-cycle checkpoints in the bacterial cell, the arrest of the septation process does not immediately arrest cell elongation and replication (*30*). Growth arrest is therefore detected first by the cell-number metric, directly dependent on septation, and later in the other two metrics. The genome-wide TFNseq approach measures replication-based arrest and is therefore expected to be most precise for replication genes and overestimate the arrest time for other processes. In spite of this limitation, the TFNseq approach is tractable at a genomic scale and is accurate enough to capture the transcription-based trend predicted by the RLTO model (Fig. 4A).

### Other determinants of overabundance

In addition to these experimental limitations, the RLTO model itself relies on a number of important simplifications. Several authors have already focused on the significance of protein function, which appears to play a central role in shaping the fitness landscape (*9*, *18*, *31*). Here, we will focus on exploring two specific function-related hypotheses that affect RLTO model predictions (*11*): the role of gene regulation and protein toxicity in determining overabundance.

### Tightly regulated genes have low overabundance

A key assumption in the RLTO model is that gene expression noise is a consequence of the number of mRNA only and is otherwise independent of regulation (*11*). Precise control of expression could lead to a reduction in the optimal overabundance. To explore the regulatory hypothesis, we generated three lists of essential genes: autoregulatory, highly regulated (top 10% of genes ranked by the number of regulators), and unregulated. We classified essential genes on the basis of their annotated regulatory relationships in the fellow Gammaproteobacteria *E. coli* whose regulome has been much more extensively characterized than *A. baylyi*. If regulation can obviate the need for overabundance, we would expect lower median overabundances in both regulated groups and potentially higher overabundances for the unregulated group relative to all essential genes. Consistent with this hypothesis, we find that the median overabundance for autoregulatory genes is onefold and, for highly regulated genes, threefold compared with sevenfold for all essential genes and 12-fold for un-regulated genes, supporting the hypothesis that tight regulation could reduce the need for overabundance (see Fig. 4B). We emphasize that the literature annotations are incomplete and unannotated regulation could explain genes that have both low overabundance and an unregulated annotation.

### Proteins with functional potential for toxicity have low overabundance

A second key assumption in the RLTO model is that the metabolic costs of transcription and translation are the dominant fitness costs of protein overabundance (i.e., there is no toxicity) (*11*). To explore the potential role of toxicity, we generated groups of essential adenosine triphosphatases (ATPases) and enzymes, hypothesizing that these proteins would have higher cost because of excessive activity when overabundant, and a group of DNA binding proteins, which we hypothesized would have low cost when overabundant. We find that the median overabundance for ATPase genes is twofold and, for enzymes, more generally fivefold compared to sevenfold for all essential genes and 13-fold for DNA binding proteins. These results are consistent with the hypothesis that toxicity, and in particular ATPase activity, is also a key determinant of overabundance (see Fig. 4B). We note that other authors have already focused on describing more protein function–centric analyses, which demonstrate protein overabundance for protein in metabolism and other processes [e.g., (*8*, *9*, *18*, *31*)].

## DISCUSSION

### Shape of the fitness landscape

Despite some large-scale measurements (*8*, *9*, *31*–*33*), fundamental questions remain about the structure of the fitness landscape and its rationale (*7*). Our genome-wide measurements reveal that most (69%) essential proteins are consistent with a step-like transition between wild-type and arrested growth below a critical threshold protein abundance. These step-like transitions are qualitatively consistent with asymmetric landscapes that have been observed previously, which transition more gradually from the arrest of slow growth to a relatively flat plateau [e.g., (*3*, *31*, *32*)]. Does the detailed form of the fitness landscape affect RLTO predictions? It is important to emphasize that the optimality of overabundance does not depend on the detailed mathematical form of the fitness landscape: It is the strong asymmetry of that landscape that is required to predict protein overabundance (*11*).

How do we reconcile the sharpness of the transition between growth and no growth in the single-cell image-based analysis with previous measurements that show a more complex transition? An intriguing possibility is that cell-to-cell variation in protein abundance may obscure the underlying landscape. In imaging-based experiments, once protein expression ceases, the remaining proteins are partitioned between daughter cells with very low noise (*24*). A sharp transition is therefore expected in the progeny that begins from a common pool of proteins in a single progenitor cell (Fig. 1A). We hypothesize that the observed progenitor-to-progenitor variation in the arrest time is due to differences in the single-cell protein abundances in the progenitor cells (Fig. 2C). As previously discussed (*16*), steady-state CRISPRi-based protein depletion may increase cell-to-cell variation in protein expression beyond the existing endogenous expression noise, further smoothing the underlying fitness landscape. To our knowledge, there has yet to be any consistent analysis of noise in CRISPRi silencing in bacterial cells, even though this phenomenon could clearly limit the resolution of steady-state CRISPRi-based experiments. That said, the knockout-depletion approach or analogous CRISPRi-based dilution approaches (*9*) are not a steady-state measurement of cell growth, and therefore, this dilution-based approach to measuring the fitness landscape may introduce its own systematic limitations.

### Rationale for a threshold abundance

**Fig. 5.**
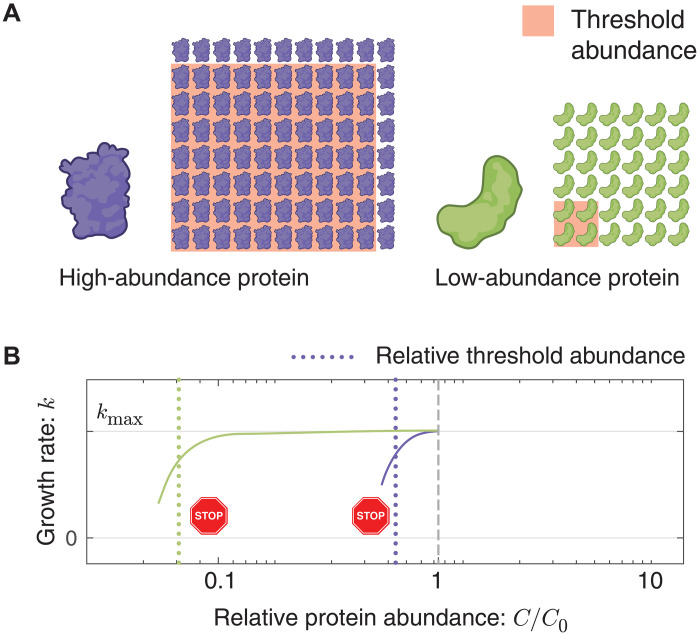
How rate-limited kinetics shapes the fitness landscape. (**A**) Protein abundance and threshold. Two essential protein species with different abundances are pictured schematically. The threshold abundance at which each protein becomes limiting is represented by the pink square, and the total cellular abundance is represented by the protein array. (**B**) Emergent fitness landscape. A schematic model of the growth rate versus relative protein abundance is shown for the two protein species. The RLTO model predicts that low-abundance proteins (green) have high overabundance, which leads to insensitivity to protein depletion. High-abundance proteins (purple) are predicted to have small overabundance, which leads to high sensitivity to protein dilution. The growth rate rapidly decreases with concentration once a species becomes limiting.

The observed threshold-like dependence can be rationalized in terms of chemical kinetics: Changing the abundance of a reactant in a multicomponent reaction will only change the rate if the reactant is rate-limiting (*11*, *34*). As the abundance of the rate limiting reactant *i* is increased, the reaction rate also increases until another reactant becomes rate-limiting, after which the rate saturates with respect to *i*, giving rise to a threshold (see Fig. 5). We therefore hypothesize that the threshold-like behavior observed in the fitness landscape is the consequence of nearly all proteins being expressed in excess of the rate-limiting concentration. Consistent with this picture, we explicitly demonstrate that the protein function (i.e., replication) is robust to an order-of-magnitude depletion of replisome protein DnaN; however, for most proteins, we must infer this picture from the growth rate.

### Rationale for overabundance

Rate-limiting kinetics does not in itself predict vast protein overabundance. The RLTO model predicts that this feature of the fitness landscape is a consequence of a balance between (i) the metabolic cost of protein expression, which favors minimizing protein abundance, and (ii) robustness to the noise in gene expression (*35*, *36*). The model predicts expression-dependent protein overabundance: large overabundance for low-abundance proteins and small overabundance for high-abundance proteins resulting from the highly asymmetric fitness trade-off; the metabolic cost of overabundance for low-expression genes is small compared with the failure of essential processes (*11*). We show that this signature prediction is observed (Fig. 4A).

### Environmental fluctuations

The overabundance predicted by the RLTO model hedges against noise, i.e., the cell-to-cell variation in gene expression; however, it has also been proposed that overabundance might result from hedging against a second form of fluctuations, environmental fluctuations, in which the cell transition from one carbon source to another or from good to poor growth conditions (*37*, *38*). In other words, is overabundance the result of simultaneously optimizing for multiple objectives? We do not reject this hypothesis outright, especially in the context of genes involved in metabolism; however, the mechanism we propose is much more generic and predicts that overabundance should be optimal even for low-expression proteins with little direct connection to metabolism (e.g., replication, cell-wall synthesis, etc.).

### Is protein overabundance conserved?

To what extent is the overabundance of essential genes a conserved mechanism from bacteria to single-cell eukaryotes and to multicellular organisms? If overabundance were specific to *A. baylyi*, we would expect marked differences between the *A. baylyi* and *E. coli* transcriptomes for low-expression essential-gene homologs. This is not observed (fig. S11). As we emphasized above, CRISPRi protein depletions in a wide range of model organisms appear to be qualitatively consistent with the overabundance hypothesis in that large-factor depletions are often required to generate a phenotype (*8*, *12*–*15*). Furthermore, we have demonstrated elsewhere that the RLTO model also predicts two other principles of central dogma function that are observed (*11*): (i) The one-message rule defines a lower limit on transcription, below which gene expression noise becomes too high for robust growth. At the threshold transcription level, cell-to-cell variation approaches the mean expression level (*11*). (ii) Transcription-translation load balancing refers to a regulatory strategy observed in eukaryotic cells, where noise is reduced by increasing transcription while decreasing the translation efficiency of low-expression genes (*11*). Because of the success of the RLTO model in predicting these other principles of central dogma function in other organisms, we expect that the overabundance strategy will also be conserved for low-expression genes.

### Are nonessential proteins overabundant?

We have focused our analysis on essential genes in the model organism *A. baylyi* and demonstrated that most essential proteins are overabundant. To what extent is this mechanism generic to nonessential proteins? Several arguments support a generic applicability to nonessential genes. Our modeling suggests that asymmetry rather than explicit growth arrest is the mathematical rationale for the optimality of overabundance (*11*). We therefore predict that all proteins that increase cell fitness, not just essential proteins, will be overabundant. In addition, it is important to emphasize that the annotation of genes as essential is contextual. For instance, for *E. coli* proliferation on lactose, the gene *lacZ* is essential, although nonessential for other carbon sources. As a result, we predict that when induced, LacZ should be overabundant, consistent with a previous observation (*37*). Last, the RLTO model also correctly predicts the balance between transcription and translation for all genes, not just essential genes, in eukaryotic cells, suggesting that it should generalize to nonessential genes as well (*11*).

### Biological implications

Many important proposals have been made about the biological implications of noise (*39*). Our work reveals that noise acts to inflate the optimal expression levels of low-expression proteins and, as a result, significantly increases the metabolic budget for protein, which constitutes 50 to 60% of the dry mass of the cell (*4*). We believe that this increased protein budget has cellular-scale implications. For instance, in the stress response and stationary phase, the presence of a reservoir of overabundant proteins provides critical resources, via protein catabolism, to facilitate the adaptation to changing conditions (*40*–*42*). Protein overabundance may have important implications for individual biological processes as well, including determining which proteins and cellular processes make attractive targets for small-molecule inhibitors (e.g., antibiotics) (*33*). Given that overabundance defines the fold depletion in protein activity required to achieve growth arrest, high-overabundance proteins are predicted to be extremely difficult targets for inhibition.

By combining imaging-, genomic-, and modeling-based approaches, we provide both a quantitative measurement of the fitness landscape for all essential proteins and clear qualitative and conceptual understanding of the rationale for the observed fitness landscape. The RLTO model fundamentally reshapes our understanding of the rationale for protein abundance. The model predicts, and experiments confirm, that low-abundance proteins are expressed in vast excess of what is required for growth. Despite the limitations of the experiments, the predicted trend is clearly resolved both at a genomic-scale, using sequencing-based approaches, and at the single-cell scale, as observed by microscopy. The rationale for the overabundance strategy is intuitive: Growth requires the robust expression of hundreds of distinct proteins. The cell contends with this extraordinary complex regulatory challenge by keeping all but the highest-abundance proteins in vast excess.

## MATERIALS AND METHODS

### *A. baylyi* strains, manipulation, and culturing

Mutant strains were derived from *A. baylyi* ADP1 (MAY101) (the gift of C. Manoil) (*43*). Growth media were LB and M9, a minimal-succinate M9 medium (*44*), supplemented with 15 mM sodium succinate, 2 mM magnesium sulfate, 0.1 mM calcium chloride, and 1 to 3 M ferrous sulfate (from sterile 5 mM stock, made fresh at least once a month). For selective growth, media were supplemented with kanamycin at 20 μg/ml. Cultures were grown at 30°C. The strains used in the study are summarized in table S2.

#### Construction of deletion mutations

We generated deletion mutants by transformation of linear DNA fragments, constructed by polymerase chain reaction (PCR) using extension overlap (*17*). A homologous overlap of ~2-kb flanking target genes was created that either directly joined (for marker-free deletions) or flanked a kanamycin resistance cassette (for kan-selectable deletions). Unmarked deletions were in-frame. Kan deletions were constructed from the *kan* gene from plasmid pACYC177 (*45*) in an orientation matching the deleted gene (*17*). PCRs were performed using Q5 Polymerase (New England Biolabs) or Phusion HF polymerase (New England Biolabs), and DNA fragments were purified using Qiaquick columns (Qiagen) before transformation.

#### A. baylyi transformation protocol

DNA fragments were transformed into *A. baylyi* cultures prepared as follows. Cultures were grown overnight in minimal-succinate M9 media with 1 μM ferrous sulfate. The culture was then diluted 1:5 into fresh medium and grown for 1 hour, with shaking at 30°C. The DNA fragment was added at 1 μg/ml, followed by incubation for 2.5 to 3 hours with shaking, and then plated on selective (for *kan*-deletion cassettes) or nonselective media (for marker-free cassettes). Marker-free deletion mutants were identified by screening single colonies by PCR using primers flanking targeted genes. Essential gene kan-marked deletion mutations were selected by plating on protective medium supplemented with kanamycin (20 μg/ml). All unmarked and marked nonessential deletion mutations were verified by PCR. For essential gene deletions, 0.1 to 1% of the cells were transformed, forming microcolonies of cells carrying the deletion.

#### Construction of YPet-dnaN fusion strain

In previous work in *E. coli* and *Bacillus subtilis*, we visualized fluorescent fusions to the β sliding clamp (*dnaN*) to study replication (*21*–*23*). DnaN protein imaging is a convenient tool for studying replication because of its relatively high abundance and the change in its localization from diffuse (nonreplicating cells) to punctate (replicating cells), which serves as a convenient reporter of activity. To construct a fluorescent fusion to the *A. baylyi* DnaN protein with a high probability of success, we used the exact same fluorescent protein and linker that R. Reyes-Lamothe used to construct the *E. coli* fusion used in our previous work (*27*). In this approach, we inserted the YPet-linker cassette at the 5′ end of the gene. Given that the transformation efficiency of *A. baylyi* is so high, we constructed a marker-free fusion. We screened and confirmed the YdnaN colonies by both PCR and fluorescence localization. Like the original *E. coli* strain, no growth phenotype is observed under experimental conditions.

### Imaging-based knockout-depletion experiments

For single-cell imaging-based analyses, cells were imaged proliferating in M9 media supplemented with 2% low-melt agarose and, in most cases, kanamycin at 20 μg/ml.

#### Cell preparation for knockout-depletion experiments

The transformation protocol described above was modified as follows: After the 2.5- to 3-hour incubation with DNA, cells were immediately spotted on selective medium pads for imaging. In the knockout-depletion experiments, cells are transformed with knockout cassettes, which recombine into the genome, resulting in Km^R^ knockout strains. If transformed cells are transferred to Km^+^ media too quickly, the competent cells do not have sufficient time to integrate the *kan* cassette before growth arrest. If cells are transferred too late, essential proteins are depleted before imaging begins. How do we know that transformants after 2.5- to 3-hour outgrowth are at their initial stages of transient growth? With the 2.5- to 3-hour outgrowth period, many cells still grow slowly (compared to log-phase growth) for 10 to 15 min, consistent with the expression of the kanamycin phosphotransferase (the gene product of the *kan* gene) not having reached a sufficiently high level to achieve a resistance phenotype. Furthermore, a number of heterogenic progenitors were observed. The presence of these heterogenic progenitor cells is consistent with the 2.5-hour outgrowth period representing the typical recombination time for transformants (see section S1A for a discussion of heterogenic progenitors).

#### Sample and slide preparation

Thin pads were fabricated by melting the agarose (Invitrogen UltraPure LMP Agarose) and casting it between two slides with two layers of lab tape used as a shim to set the height. After the pad solidified (roughly 10 min), the top slide was carefully removed, and a razor blade was used to trim the pad to form a small square that could be covered with a #1.5 coverslip. For *E. coli* imaging, we typically use a pad that matches the size of the coverslip; however, for *A. baylyi* imaging, we trim the pad so it is less than 1 cm in width. This added space allows aerobic growth to continue over multiple hours. Last, the coverslip is sealed using a hot glue gun.

#### Microscopy

The samples were imaged using a Nikon Eclipse Ti microscope in phase contrast and fluorescence. We imaged through a Nikon 60×/1.4–numerical aperture phase contrast objective onto a scientific complementary metal-oxide semiconductor camera (Andor Neo). An environmental chamber maintained the sample at 30°C during imaging. For phase imaging, a frame rate of 1 frame/2 min was used; however, for combined phase and fluorescence imaging, we reduced the frame rate to 1 frame/3 min and 1 frame/9 min to help reduce bleaching and phototoxicity. (The slowest frame rate was used to resolve the dim YPet-DnaN foci as the protein levels were depleted.) Typically, multiple (~10) fields of view were captured simultaneously in each experiment. For fluorescence-based analysis, we mixed in wild-type cells, in addition to fluorescent-fusion cells (1:2), to determine the autofluorescence levels in each experiment.

## Supplementary Materials

### The PDF file includes:

Supplementary Text Figs. S1 to S14 Tables S1 to S3 Legends for data S1 to S9 Legends for movies S1 to S12 References

## References

[1] B. Alberts, A. Johnson, J. Lewis, M. Raff, K. Roberts, P. Walter, *Molecular Biology of the Cell* (Garland, ed. 4, 2002).

[2] E. Dekel, U. Alon, Optimality and evolutionary tuning of the expression level of a protein. Nature 436, 588–592 (2005). 16049495 10.1038/nature03842

[3] J.-B. Lalanne, D. J. Parker, G.-W. Li, Spurious regulatory connections dictate the expression-fitness landscape of translation factors. Mol. Syst. Biol. 17, e10302 (2021). 33900014 10.15252/msb.202110302 PMC8073009

[4] J. W. Lengeler, G. Drews, H. G. Schlegel, Eds., *Biology of the Prokaryotes* (Georg Thieme Verlag, 1998); 10.1002/9781444313314.

[5] M. Kafri, E. Metzl-Raz, F. Jonas, N. Barkai, Rethinking cell growth models. FEMS Yeast Res. 16, fow081 (2016). 27650704 10.1093/femsyr/fow081

[6] J. Hausser, A. Mayo, L. Keren, U. Alon, Central dogma rates and the trade-off between precision and economy in gene expression. Nat. Commun. 10, 68 (2019). 30622246 10.1038/s41467-018-07391-8 PMC6325141

[7] N. M. Belliveau, G. Chure, C. L. Hueschen, H. G. Garcia, J. Kondev, D. S. Fisher, J. A. Theriot, R. Phillips, Fundamental limits on the rate of bacterial growth and their influence on proteomic composition. Cell Syst. 12, 924–944.e2 (2021). 34214468 10.1016/j.cels.2021.06.002 PMC8460600

[8] J. M. Peters, A. Colavin, H. Shi, T. L. Czarny, M. H. Larson, S. Wong, J. S. Hawkins, C. H. S. Lu, B. M. Koo, E. Marta, A. L. Shiver, E. H. Whitehead, J. S. Weissman, E. D. Brown, L. S. Qi, K. C. Huang, C. A. Gross, A comprehensive, CRISPR-based functional analysis of essential genes in bacteria. Cell 165, 1493–1506 (2016). 27238023 10.1016/j.cell.2016.05.003 PMC4894308

[9] S. Donati, M. Kuntz, V. Pahl, N. Farke, D. Beuter, T. Glatter, J. V. Gomes-Filho, L. Randau, C. Y. Wang, H. Link, Multi-omics analysis of CRISPRi-knockdowns identifies mechanisms that buffer decreases of enzymes in E. coli metabolism. Cell Syst. 12, 56–67.e6 (2021). 33238135 10.1016/j.cels.2020.10.011

[10] T. Baba, H.-C. Huan, K. Datsenko, B. L. Wanner, H. Mori, The applications of systematic in-frame, single-gene knockout mutant collection of *Escherichia coli* K-12. Methods Mol. Biol. 416, 183–194 (2008). 18392968 10.1007/978-1-59745-321-9_12

[11] T. W. Lo, H. J. Choi, D. Huang, P. A. Wiggins, Noise robustness and metabolic load determine the principles of central dogma regulation. Sci. Adv. 10, eado3095 (2024). 39178264 10.1126/sciadv.ado3095 PMC11343026

[12] K. E. McGinness, T. A. Baker, R. T. Sauer, Engineering controllable protein degradation. Mol. Cell 22, 701–707 (2006). 16762842 10.1016/j.molcel.2006.04.027

[13] J. H. Davis, T. A. Baker, R. T. Sauer, Small-molecule control of protein degradation using split adaptors. ACS Chem. Biol. 6, 1205–1213 (2011). 21866931 10.1021/cb2001389 PMC3220803

[14] D. E. Cameron, J. J. Collins, Tunable protein degradation in bacteria. Nat. Biotechnol. 32, 1276–1281 (2014). 25402616 10.1038/nbt.3053 PMC4262603

[15] X. Liu, C. Gallay, M. Kjos, A. Domenech, J. Slager, S. P. van Kessel, K. Knoops, R. A. Sorg, J.-R. Zhang, J.-W. Veening, High-throughput CRISPRi phenotyping identifies new essential genes in *Streptococcus pneumoniae*. Mol. Syst. Biol. 13, 931 (2017). 28490437 10.15252/msb.20167449 PMC5448163

[16] S. N. J. Franks, R. Heon-Roberts, B. J. Ryan, CRISPRi: A way to integrate iPSC-derived neuronal models. Biochem. Soc. Trans. 52, 539–551 (2024). 38526223 10.1042/BST20230190 PMC11088925

[17] J. Bailey, J. Cass, J. Gasper, N.-D. Ngo, P. Wiggins, C. Manoil, Essential gene deletions producing gigantic bacteria. PLOS Genet. 15, e1008195 (2019). 31181062 10.1371/journal.pgen.1008195 PMC6586353

[18] L. A. Gallagher, J. Bailey, C. Manoil, Ranking essential bacterial processes by speed of mutant death. Proc. Natl. Acad. Sci. U.S.A. 117, 18010–18017 (2020). 32665440 10.1073/pnas.2001507117 PMC7395459

[19] K. T. Elliott, E. L. Neidle, *Acinetobacter baylyi* ADP1: Transforming the choice of model organism. IUBMB Life 63, 1075–1080 (2011). 22034222 10.1002/iub.530

[20] D. Metzgar, J. M. Bacher, V. Pezo, J. Reader, V. Döring, P. Schimmel, P. Marlière, V. de Crécy-Lagard, *Acinetobacter sp.* ADP1: An ideal model organism for genetic analysis and genome engineering. Nucleic Acids Res. 32, 5780–5790 (2004). 15514111 10.1093/nar/gkh881 PMC528786

[21] S. M. Mangiameli, C. N. Merrikh, P. A. Wiggins, H. Merrikh, Transcription leads to pervasive replisome instability in bacteria. eLife 6, e19848 (2017). 28092263 10.7554/eLife.19848 PMC5305214

[22] S. M. Mangiameli, B. T. Veit, H. Merrikh, P. A. Wiggins, The replisomes remain spatially proximal throughout the cell cycle in bacteria. PLOS Genet. 13, e1006582 (2017). 28114307 10.1371/journal.pgen.1006582 PMC5293282

[23] S. M. Mangiameli, J. A. Cass, H. Merrikh, P. A. Wiggins, The bacterial replisome has factory-like localization. Curr. Genet. 64, 1029–1036 (2018). 29632994 10.1007/s00294-018-0830-z

[24] N. J. Kuwada, B. Traxler, P. A. Wiggins, Genome-scale quantitative characterization of bacterial protein localization dynamics throughout the cell cycle. Mol. Microbiol. 95, 64–79 (2015). 25353361 10.1111/mmi.12841 PMC4309519

[25] G. M. Cooper, *The Cell: A Molecular Approach* (Sinauer Associates 2000, ed. 2, 2000).

[26] K. P. Lemon, A. D. Grossman, Localization of bacterial dna polymerase: Evidence for a factory model of replication. Science 282, 1516–1519 (1998). 9822387 10.1126/science.282.5393.1516

[27] R. Reyes-Lamothe, D. J. Sherratt, M. C. Leake, Stoichiometry and architecture of active DNA replication machinery in *Escherichia coli*. Science 328, 498–501 (2010). 20413500 10.1126/science.1185757 PMC2859602

[28] K. J. Cutler, C. Stringer, T. W. Lo, L. Rappez, N. Stroustrup, S. Brook Peterson, P. A. Wiggins, J. D. Mougous, Omnipose: A high-precision morphology-independent solution for bacterial cell segmentation. Nat. Methods 19, 1438–1448 (2022). 36253643 10.1038/s41592-022-01639-4 PMC9636021

[29] V. de Berardinis, D. Vallenet, V. Castelli, M. Besnard, A. Pinet, C. Cruaud, S. Samair, C. Lechaplais, G. Gyapay, C. Richez, M. Durot, A. Kreimeyer, F. L. Fèvre, V. Schächter, V. Pezo, V. Döring, C. Scarpelli, C. Médigue, G. N. Cohen, P. Marlière, M. Salanoubat, J. Weissenbach, A complete collection of single-gene deletion mutants of *Acinetobacter baylyi* ADP1. Mol. Syst. Biol. 4, 174 (2008). 18319726 10.1038/msb.2008.10 PMC2290942

[30] S. Autret, A. Levine, I. B. Holland, S. J. Séror, Cell cycle checkpoints in bacteria. Biochimie 79, 549–554 (1997). 9466691 10.1016/s0300-9084(97)82002-0

[31] J. S. Hawkins, M. R. Silvis, B. M. Koo, J. M. Peters, H. Osadnik, M. Jost, C. C. Hearne, J. S. Weissman, H. Todor, C. A. Gross, Mismatch-CRISPRi reveals the co-varying expression-fitness relationships of essential genes in *Escherichia coli* and *Bacillus subtilis*. Cell Syst. 11, 523–535.e9 (2020). 33080209 10.1016/j.cels.2020.09.009 PMC7704046

[32] L. Keren, J. Hausser, M. Lotan-Pompan, I. Vainberg Slutskin, H. Alisar, S. Kaminski, A. Weinberger, U. Alon, R. Milo, E. Segal, Massively parallel interrogation of the effects of gene expression levels on fitness. Cell 166, 1282–1294.e18 (2016). 27545349 10.1016/j.cell.2016.07.024

[33] B. Bosch, M. DeJesus, N. C. Poulton, W. Zhang, C. A. Engelhart, A. Zaveri, S. Lavalette, N. Ruecker, C. Trujillo, J. B. Wallach, S. Li, S. Ehrt, B. T. Chait, D. Schnappinger, J. M. Rock, Genome-wide gene expression tuning reveals diverse vulnerabilities of *M. tuberculosis*. Cell 184, 4579–4592.e24 (2021). 34297925 10.1016/j.cell.2021.06.033 PMC8382161

[34] D. L. Nelson, M. M. Cox, *Lehninger Principles of Biochemistry* (W.H. Freeman, ed. 7, 2017).

[35] J. Paulsson, M. Ehrenberg, Random signal fluctuations can reduce random fluctuations in regulated components of chemical regulatory networks. Phys. Rev. Lett. 84, 5447–5450 (2000). 10990965 10.1103/PhysRevLett.84.5447

[36] N. Friedman, L. Cai, X. S. Xie, Linking stochastic dynamics to population distribution: An analytical framework of gene expression. Phys. Rev. Lett. 97, 168302 (2006). 17155441 10.1103/PhysRevLett.97.168302

[37] G. Lambert, E. Kussell, Memory and fitness optimization of bacteria under fluctuating environments. PLOS Genet. 10, e1004556 (2014). 25255314 10.1371/journal.pgen.1004556 PMC4177670

[38] M. Mori, S. Schink, D. W. Erickson, U. Gerland, T. Hwa, Quantifying the benefit of a proteome reserve in fluctuating environments. Nat. Commun. 8, 1225 (2017). 29089487 10.1038/s41467-017-01242-8 PMC5663898

[39] J. M. Raser, E. K. O’Shea, Noise in gene expression: Origins, consequences, and control. Science 309, 2010–2013 (2005). 16179466 10.1126/science.1105891 PMC1360161

[40] D. Weichart, N. Querfurth, M. Dreger, R. Hengge-Aronis, Global role for ClpP-containing proteases in stationary-phase adaptation of *Escherichia coli*. J. Bacteriol. 185, 115–125 (2003). 12486047 10.1128/JB.185.1.115-125.2003 PMC141834

[41] A. L. Goldberg, A. C. St John, Intracellular protein degradation in mammalian and bacterial cells: Part 2. Annu. Rev. Biochem. 45, 747–803 (1976). 786161 10.1146/annurev.bi.45.070176.003531

[42] M. Gupta, A. N. T. Johnson, E. R. Cruz, E. J. Costa, R. L. Guest, S. H.-J. Li, E. M. Hart, T. Nguyen, M. Stadlmeier, B. P. Bratton, T. J. Silhavy, N. S. Wingreen, Z. Gitai, M. Wühr, Global protein turnover quantification in *Escherichia coli* reveals cytoplasmic recycling under nitrogen limitation. Nat. Commun. 15, 5890 (2024). 39003262 10.1038/s41467-024-49920-8 PMC11246515

[43] V. Barbe, D. Vallenet, N. Fonknechten, A. Kreimeyer, S. Oztas, L. Labarre, S. Cruveiller, C. Robert, S. Duprat, P. Wincker, L. N. Ornston, J. Weissenbach, P. Marlière, G. N. Cohen, C. Médigue, Unique features revealed by the genome sequence of *Acinetobacter* sp. ADP1, a versatile and naturally transformation competent bacterium. Nucleic Acids Res. 32, 5766–5779 (2004). 15514110 10.1093/nar/gkh910 PMC528795

[44] J. Miller, *Experiments in Molecular Genetics* (Cold Spring Harbor Laboratory, 1972).

[45] A. C. Chang, S. N. Cohen, Construction and characterization of amplifiable multicopy DNA cloning vehicles derived from the P15A cryptic miniplasmid. J. Bacteriol. 134, 1141–1156 (1978). 149110 10.1128/jb.134.3.1141-1156.1978 PMC222365

[46] D. R. Cox, D. V. Hinkley, *Theoretical Statistics* (Chapman & Hall, 1974).

[47] T. W. Lo, K. J. Cutler, S. Stylianidou, C. Brennan, S. B. Nissen, N. J. Kuwada, P. A. Wiggins, OmniSegger, GitHub (2024); https://github.com/tlo-bot/omnisegger.

[48] S. Stylianidou, C. Brennan, S. B. Nissen, N. J. Kuwada, P. A. Wiggins, SuperSegger: Robust image segmentation, analysis and lineage tracking of bacterial cells. Mol. Microbiol. 102, 690–700 (2016). 27569113 10.1111/mmi.13486

[49] T. W. Lo, K. J. Cutler, H. J. Choi, P. A. Wiggins, Omnisegger: A time-lapse image analysis pipeline for bacterial cells. bioRxiv 625259 [Preprint] (2024). 10.1101/2024.11.25.625259. PMC12140430 40435362

[50] K. P. Burnham, D. R. Anderson, Multimodel inference: Understanding AIC and BIC in model selection. Social. Methods Res. 33, 261–304 (2004).

[51] P. D. Karp, S. Paley, R. Caspi, A. Kothari, M. Krummenacker, P. E. Midford, L. R. Moore, P. Subhraveti, S. Gama-Castro, V. H. Tierrafria, P. Lara, L. Muñiz-Rascado, C. Bonavides-Martinez, A. Santos-Zavaleta, A. Mackie, G. Sun, T. A. Ahn-Horst, H. Choi, M. W. Covert, J. Collado-Vides, I. Paulsen, The EcoCyc database (2023). EcoSal Plus 11, eesp-0002-2023 (2023). 10.1128/ecosalplus.esp-0002-2023 PMC10729931 37220074

[52] P. H. Culviner, C. K. Guegler, M. T. Laub, A simple, cost-effective, and robust method for rRNA depletion in RNA-sequencing studies. mBio 11, e00010–e00020 (2020). 32317317 10.1128/mBio.00010-20 PMC7175087

[53] Y. Taniguchi, P. J. Choi, G. W. Li, H. Chen, M. Babu, J. Hearn, A. Emili, X. S. Xie, Quantifying *E. coli* proteome and transcriptome with single-molecule sensitivity in single cells. Science 329, 533–538 (2010). 20671182 10.1126/science.1188308 PMC2922915

[54] F. Crick, Central dogma of molecular biology. Nature 227, 561–563 (1970). 4913914 10.1038/227561a0

[55] J. L. Hargrove, F. H. Schmidt, The role of mRNA and protein stability in gene expression. FASEB J. 3, 2360–2370 (1989). 2676679 10.1096/fasebj.3.12.2676679

[56] A. L. Koch, H. R. Levy, Protein turnover in growing cultures of *Escherichia coli*. J. Biol. Chem. 217, 947–957 (1955). 13271454

[57] M. Martin-Perez, J. Villén, Determinants and regulation of protein turnover in yeast. Cell Syst. 5, 283–294.e5 (2017). 28918244 10.1016/j.cels.2017.08.008 PMC5935796

[58] M. Scott, C. W. Gunderson, E. M. Mateescu, Z. Zhang, T. Hwa, Interdependence of cell growth and gene expression: Origins and consequences. Science 330, 1099–1102 (2010). 21097934 10.1126/science.1192588

[59] E. Levien, J. Min, J. Kondev, A. Amir, Non-genetic variability in microbial populations: Survival strategy or nuisance? Rep. Prog. Phys. 84, 116601 (2021). 10.1088/1361-6633/ac2c92 34825896

[60] E. O. Powell, Growth rate and generation time of bacteria, with special reference to continuous culture. Microbiology 15, 492–511 (1956). 10.1099/00221287-15-3-492 13385433

[61] S. S. Wilks, The large-sample distribution of the likelihood ratio for testing composite hypotheses. Ann. Math. Stat. 9, 60–62 (1938).

[62] G. Casella, R. Berger, *Statistical Inference* (Duxbury Resource Center, 2001).

[63] E. W. Weisstein, *CRC Encyclopedia of Mathematics* (Chapman & Hall/CRC, 2009).

