# Supplementary material for: Protein overabundance is driven by growth robustness
Source: Sci Adv. 2026 Mar 20;12(12):eadz9623. doi: 10.1126/sciadv.adz9623 (PMC13004021; doi:10.1126/sciadv.adz9623)
Supplement: Supplementary file 1 — Supplementary Text Figs. S1 to S14 Tables S1 to S3 Legends for data S1 to S9 Legends for movies S1 to S12 References [file sciadv.adz9623_sm.pdf]

Science Advances
AAAS

Supplementary Materials for

Protein overabundance is driven by growth robustness

H. James Choi *et al.*

Corresponding author: Paul A. Wiggins, pwiggins@uw.edu

Sci. Adv. **12**, eadz9623 (2026)
DOI: 10.1126/sciadv.adz9623

The PDF file includes:

Supplementary Text
Figs. S1 to S14
Tables S1 to S3
Legends for data S1 to S9
Legends for movies S1 to S12
References

Other Supplementary Material for this manuscript includes the following:

Data S1 to S9
Movies S1 to S12

1. RESULTS: IMAGING-BASED KNOCKOUT-DEPLETION EXPERIMENTS

A. Some progenitors have heterogenic progeny

Heterogenic progenitors are progenitor cells that are observed to have progeny with two distinct heritable phenotypes: the Km^{R} knockout phenotypes and the Km^{S} wild-type phenotype. For instance, in the Δ*murA* knockout-depletion experiments, progenitors were observed with one daughter whose progeny proliferated for multiple generations on Km^{+} media before lysing, the knockout phenotype, and whose other daughters proliferated for a short period but maintained wild-type morphology. The maintenance of the wild-type morphology suggested that the cells were *murA*$^{+}$ Km^{S}. How were these cells able to proliferate while other Km^{S} cells immediately arrested?

We hypothesize that since both cells had the same progenitor, recombination occurred in the mother cell, after the *murA* gene was replicated, leading to one wild-type chromosome and one Δ*murA* chromosome. The transient growth of the wild-type cells was the result of overabundance of the *kan* gene product APH(3')II being expressed before cell division in the original mother cell.

Heterogenic progenitor cells appeared frequently for *dnaN* knockout-depletion experiments, presumably because of the location of *dnaN* in the immediate vicinity of the origin, resulting in early replication. In these experiments, an additional test of the heterogenic progenitor hypothesis was possible due to the fluorescent labeling of the target protein. Cells that arrested early with the wild-type morphology showed no protein depletion; whereas cells that displayed the mutant phenotype (filamentation) showed depleted YPet-DnaN levels.

B. Transformed cells start with log-phase protein abundances

The experimental design of knockout-depletion assays (Fig. 1A) fails if cells do not start initially with wild-type protein abundances. The question is necessitated by the transformation protocol: Cells begin in stationary phase and are mixed with DNA knockout cassette. The cells are then propagated on non-selective media for 2.5-3 h. (See Materials and Methods.)

To test the design, we characterized YPet-DnaN abundance in transformed cells (at the end of out growth after transformation) and wild-type cells. We found that the protein expression levels in the two states were comparable, with the median abundance of YPet-DnaN 27% higher in transformed cells. (See Fig. S1.) This comparable expression level is to be expected since the competent cells are grown for more than two generations on the same media as log-phase cells.

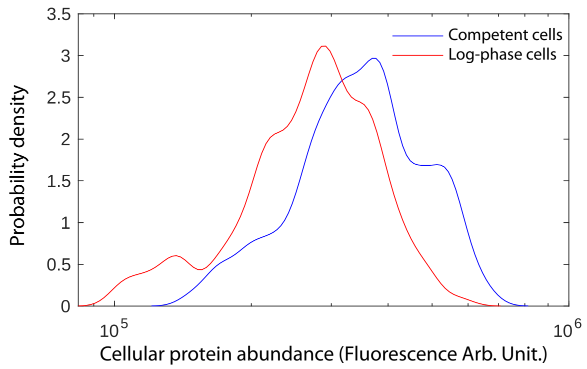

FIG. S1. **Protein abundance in competent cells is comparable to log phase.** We characterized YPet-DnaN abundance in transformed cells *before protein dilution* (at the end of out growth after transformation, $N = 115$ cells) and wild-type cells ($N = 157$ cells). The distribution of cellular protein abundances are shown above, with the median abundance of YPet-DnaN 27% higher in transformed cells.

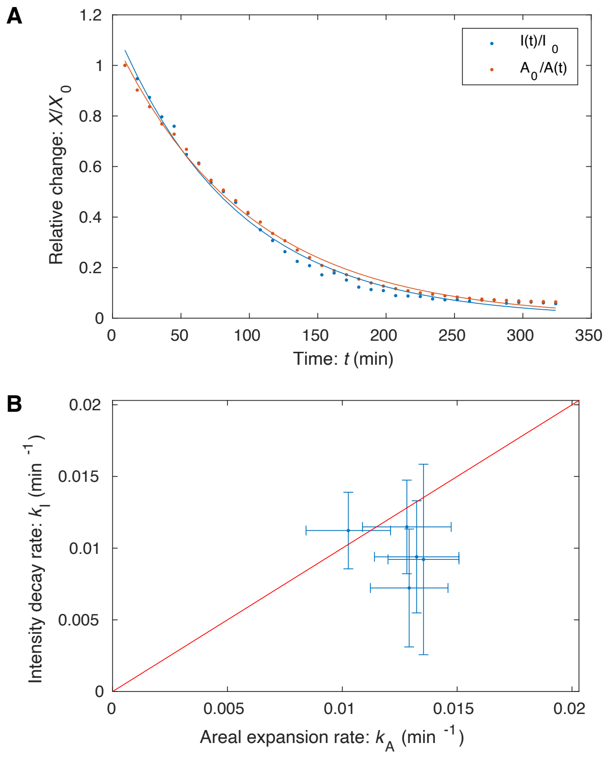

FIG. S2. **Protein is depleted by dilution. Panel A: Protein abundance and growth in a YPet-DnaN cell after knockout.** The background-subtracted average intensity and inverse area were fit to exponential functions to determine their respective rates of decay for each micro colony. **Panel B: Dilution versus growth rate.** The fluorescence decay rate k_I is compared to the areal growth rate k_A for four microcolonies. The experimental design assumes that these rate are equal. The observed rates are consistent with this assumption. The sensitivity of this experiment was limited by the fluorescence of neighboring wild-type cells.

C. Analyses of dilution rate of YPet-DnaN

The experimental design of knockout-depletion assays (Fig. 1A) fails if there is a delay before either the transformation or recombination processes. If the DNA target sequence is not rapidly degraded and expression continues, transient growth could be the consequence of this continuing expression. To test for this possibility, we determined the dilution rate of the fluorescently-labeled beta clamp (YPet-DnaN) in the YdnaN strain after knockout. If protein is depleted by dilution, the fluorescence intensity should decay with the same rate as the cell progeny area (a proxy for volume) grows (Eq. 1). In our knockout experiment, we identified four transformed progenitor cells that were isolated enough to analyze the fluorescence intensity during cell proliferation. We determined both the dilution and growth rate and compared them. (See Fig. S2A.) In each case, the rates were consistent. (See Fig. S2B.)

This experiment suffers from important limitations. Transformants are almost always adjacent to wild-type cells, as described in the last section, and fluorescence of these neighboring cells limits the sensitivity of the assay at late times due to the diffuse background generated by the brightly fluorescent wild-type cells.

D. Wild-type imaging-based analyses

We analyzed two different strains with wild-type growth phenotypes: wild-type cells (*Acinetobacter baylyi* ADP1) and ACIA0320-0321::*kan*.

IS::kan. To generate a reference wild-type growth phenotype, we choose a non-essential gene with no reported phenotype, genes ACIA0320-0321, corresponding to an IS element. The deletion was performed on the YdnaN strain, which shows no growth phenotype under the experimental conditions. We will abbreviate this strain Δ*IS*. We constructed this deletion and measured its growth relative to wild-type on Km⁻ media, and no growth phenotype was observed. However, even though this strain can be stably maintained (since ACIA0320-0321 is non-essential), we transformed this cassette using the same protocol in knockout-depletion experiments. As expected, a comparable number of transformants were observed using this construct to those targeting essential genes.

A typical transformant from a knockout-depletion experiment targeting *IS* is shown in Fig. S3 for which six generations of growth are captured. Both the areal (cell-elongation-dependent) and cell-number (septation - dependent) analyses are consistent with the null hypothesis, the *No-effect model*, as expected. The growth rate was observed to be $k = 0.925 \pm 0.005$ hr^{-1} for the areal analysis and $k = 1.04 \pm 0.14$ hr^{-1} for the cell-number

	**Area** (Cell elongation dependent)				**Cell-number** (Cell septation dependent)					
**Gene:**	Model selected:	Growth rate: k (hr^{-1})	Arrest time: T (hr)	Overabundance: $\log_{10} o$	Model selected:	Growth rate: k (hr^{-1})	Arrest time: T (hr)	Overabundance: $\log_{10} o$	Number of cells: N_C	Number of progenitors: N_P
IS(Wild-type)	No-effect	0.925 ± 0.005	NA	NA	No-effect	1.04 ± 0.14	NA	NA	60	1
dnaA	Overabundance	1.25 ± 0.02	1.2 ± 0.1	0.7 ± 0.1	Sufficiency	1.04	0.0 ± 0.3	0.0 ± 0.2	4	4
dnaN	Overabundance	1.02 ± 0.05	4.5 ± 7.7	2.0 ± 3.0	Overabundance	0.88 ± 0.07	3.8 ± 0.1	1.4 ± 0.1	134	8
ftsN	Overabundance	0.78 ± 0.06	5.2 ± 0.3	1.8 ± 0.2	Overabundance	1.12 ± 0.25	1.3 ± 0.4	0.6 ± 0.2	19	5
murA	Overabundance	0.70 ± 0.08	3.6 ± 0.4	1.1 ± 0.1	Overabundance	0.96 ± 0.24	2.0 ± 0.3	0.8 ± 0.2	29	5

TABLE S1. **Detailed results from fitting imaging-based knockout-depletion experiments.** The table summarizes the analysis of cell proliferation by two complementary metrics: area and cell-number analyses. These two metrics depend on distinct cellular processes: Growth in cell area is dependent on cell elongation, whereas the proliferation of cell number is dependent on the septation process. We give two metrics for sample size: the number of progenitors (N_P) and the total number of cells analyzed (N_C), corresponding to progenitor and progeny. The estimated standard error is provided for parameter fits.

analysis.

Qualitative phenomenology. A typical knockout-depletion experiment is shown in Fig. S3. Panel A shows a frame mosaic. The cells in this dataset show the log-phase growth phenotype of wild-type cells. Both cell number and area show exponential growth. The step-like growth of the cell number reflects the desynchronization of cell division events of the ancestors for a single progenitor.

Quantitative analysis. The null hypothesis (*Sufficiency model*) was rejected in favor of the No-effect model for both the area and cell-number analysis (both p-values under machine precision). The growth rate was observed to be $k = 1.04 \pm 0.14$ hr^{-1} for the areal analysis and $k = 0.925 \pm 0.005$ hr^{-1} for the cell-number analysis.

E. *dnaA* imaging-based analysis

Annotated gene function. DnaA is an essential regulator of the cell cycle and DNA replication initiation in particular.

Qualitative phenomenology. A typical knockout-depletion experiment is shown in Fig. S4. Panel A shows a frame mosaic. The cells in this dataset show the onset of the phenotype, cell filamentation, without undergoing significant growth-induced protein dilution. As a result, the cell number, shown in Panel B, is constant since no divisions are observed. However, as shown in Panel C, cell elongation continues for roughly 100 min before it begins to arrest. We interpret the metric that shows the earliest arrest to define the overabundance. In this case, since septation is not observed again after transformation, DnaA abundance is consistent with the Sufficiency model.

Quantitative analysis. The null hypothesis (*Sufficiency model*) was rejected in favor of the *Overabundance model* (p-value under machine precision) for the areal analysis. The initial growth rate was observed to be $k = 1.25 \pm 0.02$ hr^{-1} with an arrest time of $T = 1.24 \pm 0.10$ hr. In case of the cell number analysis, we fail to reject the null hypothesis (*Sufficiency model*), indicating that there is no statistical significance to support the alternative hypothesis *No-effect model* (p = 1.0). We used the Δ*IS* wild-type growth rate ($k = 0.925 \pm 0.005$ hr^{-1}) to fit the arrest time: $T = 0.0 \pm 0.3$ hr.

F. *dnaN* imaging-based analysis

Annotated gene function. The gene product of *dnaN* is the β sliding clamp (DnaN), which is an essential component of the replisome complex.

Qualitative phenomenology. A typical knockout-depletion experiment is shown in Fig. S5. Panel A shows a frame mosaic. The cells in this dataset show the onset of the phenotype, cell filamentation, at about 220 min, after multiple rounds of cell division. As a result, the cell number, shown in Panel B, plateaus shortly after the filamentation is observed since the filamentation is a consequence of the failure of the cells to efficiently septate. However, as shown in Panel C, cell elongation continues, although slowing slightly, throughout the experiment. In this case, since arrest is observed first with respect to septation, we use the arrest of this process to define overabundance.

Quantitative analysis. The null hypothesis (*Sufficiency model*) was rejected for both the area ($p = 8.9 \times 10^{-140}$) and cell-number analysis ($p = 6.0 \times 10^{-19}$). The initial growth rate was observed to be $k = 1.02 \pm 0.05$ hr^{-1} with an arrest time of $T = 4.5 \pm 7.7$ hr for the areal analysis. For cell-number analysis, the initial growth rate was observed to be $k = 0.88 \pm 0.07$ hr^{-1} with an arrest time of $T = 3.8 \pm 0.1$ hr.

G. *ftsN* imaging-based analysis

Annotated gene function. The gene product of *ftsN* is essential cell division protein FtsN.

Qualitative phenomenology. A typical knockout-depletion experiment is shown in Fig. S6. Panel A shows a frame mosaic. The cells in this dataset show the onset of the

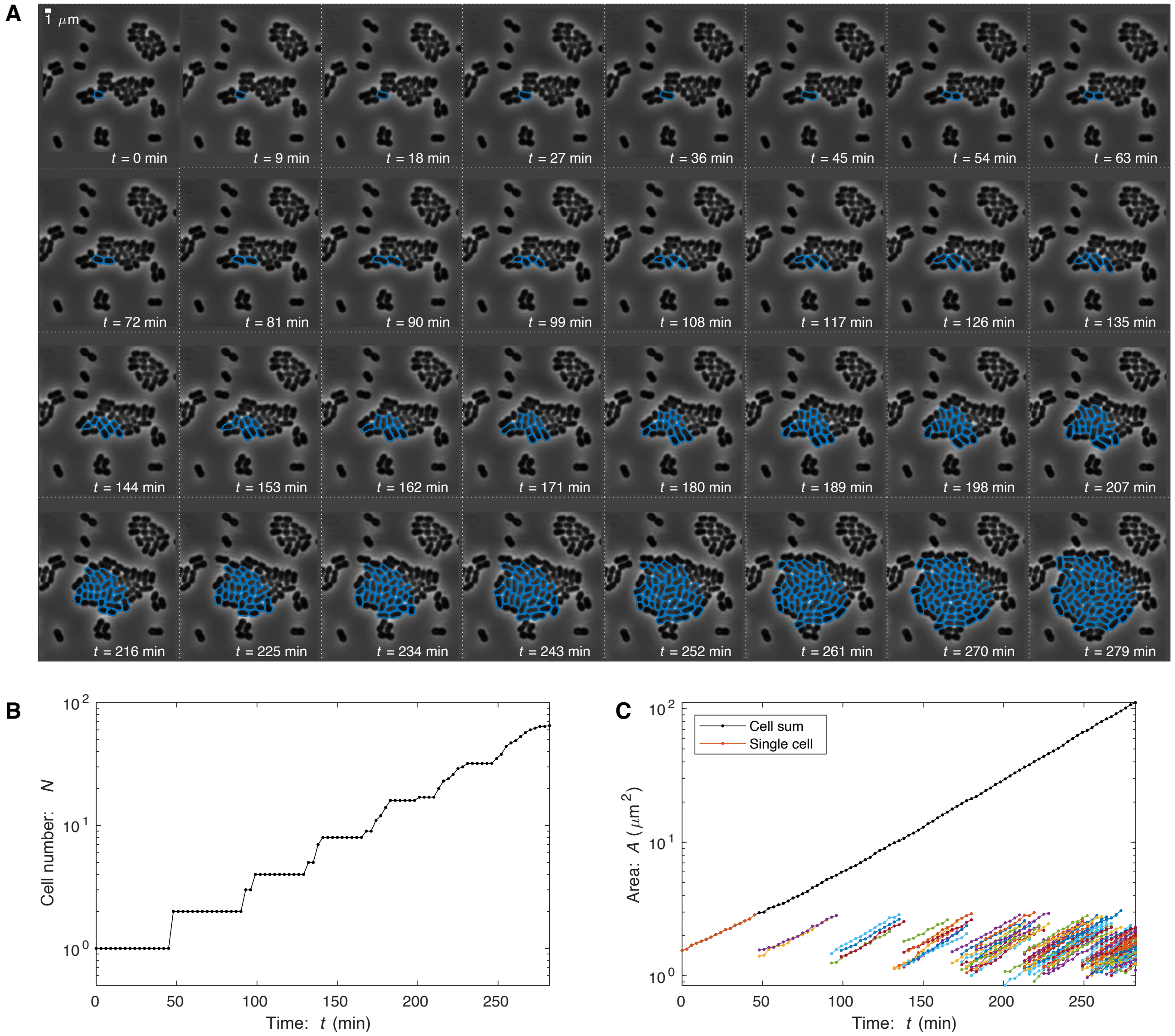

FIG. S3. **Knockout-depletion experiment: IS element (Non-essential). Panel A: Frame mosaic.** In the knockout-depletion experiment, the majority of cells are not transformed and immediately arrest on media supplemented with kanamycin. The lone transformant (*IS*::*kan* (Km^R), blue) proliferates normally. Cells were segmented using OmniSegger for quantitative analysis. **Panel B: Cell number.** The number of transformant progeny as a function of time. **Panel C: Progeny area.** Total progeny-cell area as a function of time. Total cell area is plotted with the black-dotted line, while individual cell areas are plotted with color.

phenotype: the failure to septate, at roughly 150 minutes, after several rounds of division. As a result, the cell number, shown in Panel B, plateaus shortly after 150 min as a consequence of the failure of the cells to efficiently septate. However, as shown in Panel C, cell elongation continues, although slowing slightly, to roughly 220 min.

Quantitative analysis. The null hypothesis (*Sufficiency model*) was rejected for both the area (p-value under machine precision) and cell-number analysis ($p = 1.7 \times 10^{-7}$). The initial growth rate was observed to be $k = 0.78 \pm 0.06$ hr^{-1} with an arrest time of $T = 5.2 \pm 0.3$ hr for the areal analysis. For cell-number analysis, the initial growth rate was observed to be $k = 1.12 \pm 0.25$ hr^{-1} with an arrest time of $T = 1.3 \pm 0.4$ hr.

H. *murA* imaging-based analysis

Annotated gene function. The gene product of *murA* is UDP-N-acetylglucosamine 1-carboxyvinyltransferase, an essential protein in synthesizing the precursors of cell wall synthesis.

Qualitative phenomenology. A typical knockout-depletion

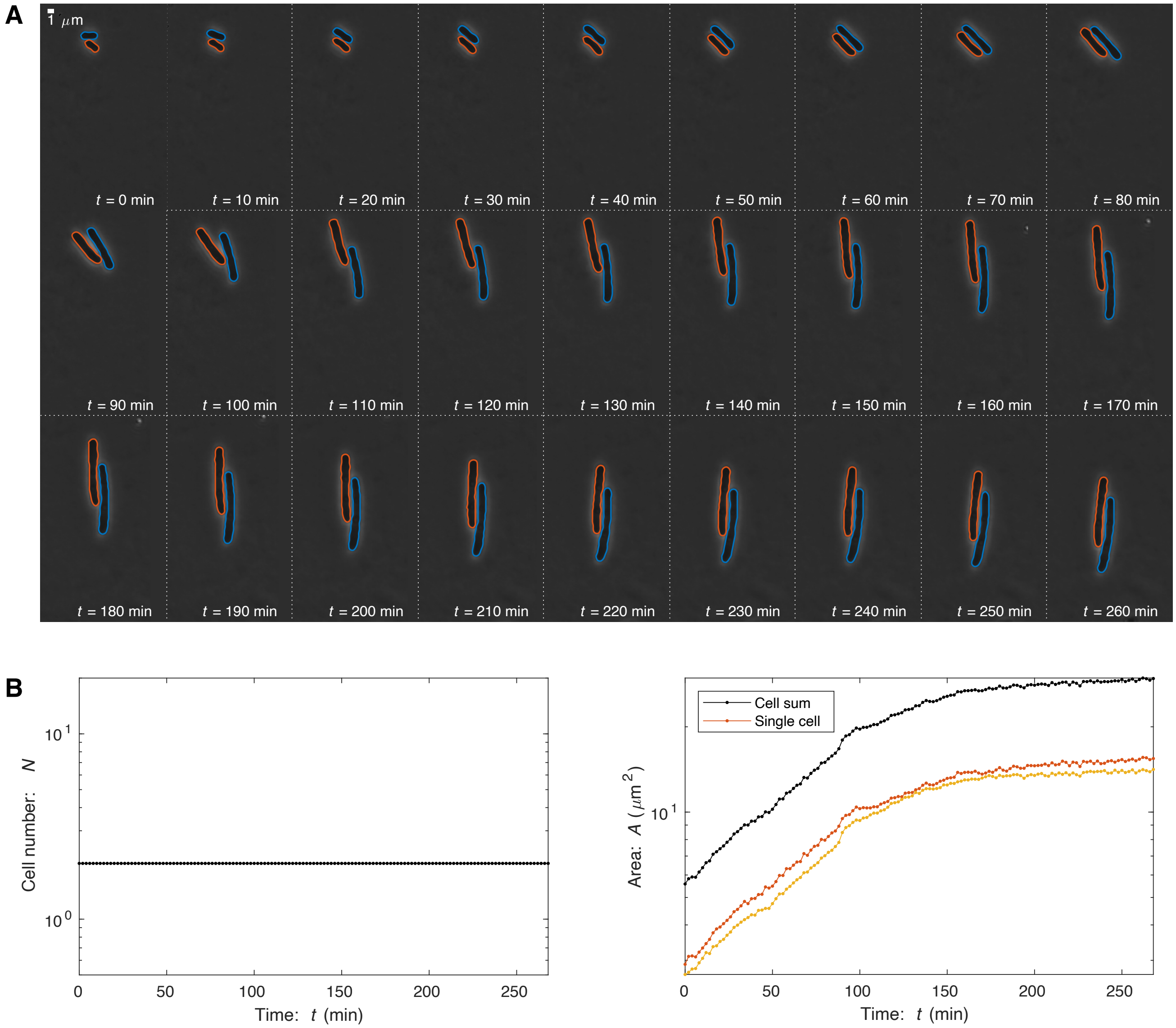

FIG. S4. **Knockout-depletion experiment:** Δ*dnaA*. **Panel A: Frame mosaic.** Two transformants (*dnaA*::*kan*(Km^R), blue, orange) proliferate. DnaA is an essential regulator of replication initiation. Its depletion leads to a failure of the chromosome to replicate, and therefore results in cell filamentation. Cells were segmented using OmniSegger for quantitative analysis. **Panel B: Cell number.** The number of transformant progeny as a function of time. After transformation, cells fail to divide, consistent with DnaA expression being sufficient rather than overabundant. **Panel C: Progeny area.** Total progeny-cell area as a function of time. In spite of the arrest of septation/division, cell areal elongation persists for roughly 120 minutes.

experiment is shown in Fig. S7. Panel A shows a frame mosaic. The cells in this dataset show the onset of the phenotype: the loss of cell wall integrity, and therefore first the loss of wild-type cell morphology and then cell lysis. Cells begin to lose their wild-type morphology at roughly 120 min, after multiple rounds of cell division. As a result, the cell number, shown in Panel B, plateaus shortly after 150 min as a consequence of the failure of the cells to efficiently septate. However, as shown in Panel C, cell elongation continues, although slowing slightly, to roughly 200 min.

Supplemental approach. For this analysis, we did not want to explicitly model cell lysis. Therefore, in our fitting of the cell-number and areal growth curves, we locked the individual cell area at the last value taken immediately preceding lysis. Similarly, we treated cells that had lysed as arrested, not absent. (This fitting-refined data is *not* shown in Fig. S7. The resulting refined data for Panels B and C plateau rather than decrease after growth arrest.)

Quantitative analysis. The null hypothesis (*Sufficiency model*) was rejected for both the area (p-value under machine precision) and cell-number analysis ($p = 1.2 \times$

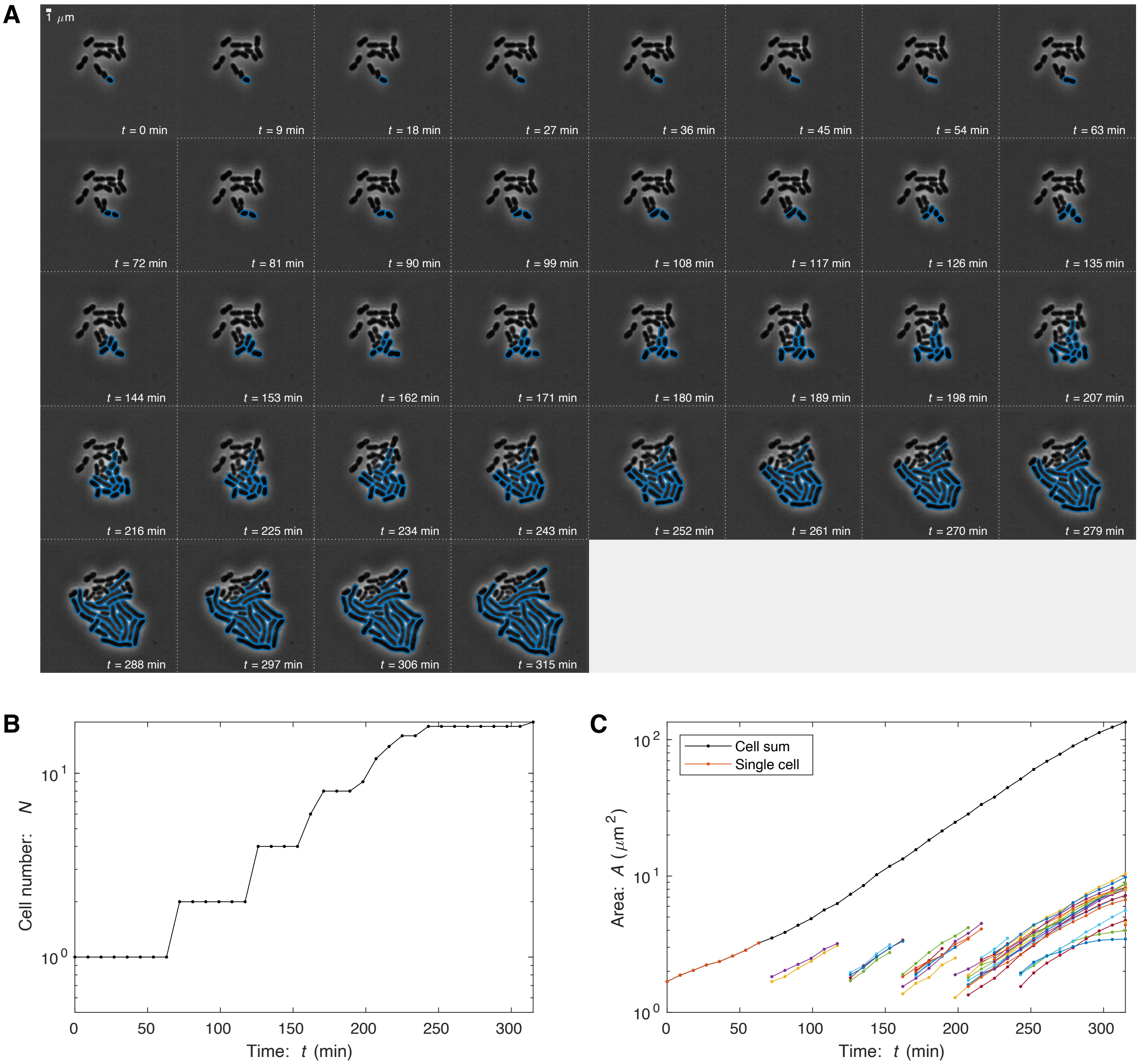

FIG. S5. **Knockout-depletion experiment:** Δ*dnaN*. **Panel A: Frame mosaic.** One transformant (*dnaN*::*kan*(Km^R), blue) proliferates. The frame mosaic shows a typical imaging-based knockout-depletion experiment. DnaN is the sliding beta divisionclamp, an essential DNA replication protein and a core component of the replisome. Its depletion leads to a failure of the chromosome to replicate and therefore results in cell filamentation. Cells were segmented using OmniSegger for quantitative analysis. **Panel B: Cell number.** The number of transformant progeny as a function of time. After transformation, normal growth persists for roughly 240 min, consistent with DnaN expression being overabundant. **Panel C: Progeny area.** Total progeny-cell area as a function of time. The areal elongation dynamics persists even after cell division arrests. (This figure shows a more detailed analysis of the data in Fig. 1.)

10^{-6}). The initial growth rate was observed to be $k = 0.70 \pm 0.08$ hr^{-1} with an arrest time of $T = 3.6 \pm 0.4$ hr for the areal analysis. For cell-number analysis, the initial growth rate was observed to be $k = 0.97 \pm 0.24$ hr^{-1} with an arrest time of $T = 2.0 \pm 0.3$ hr.

2. GROWTH MODELS FOR KNOCKOUT-DEPLETION EXPERIMENTS

To quantitatively analyze growth in knockout-depletion experiments, we define three nested growth models: (i) *No-Effect*, (ii) *Sufficiency*, and (iii) *Overabundance* models.

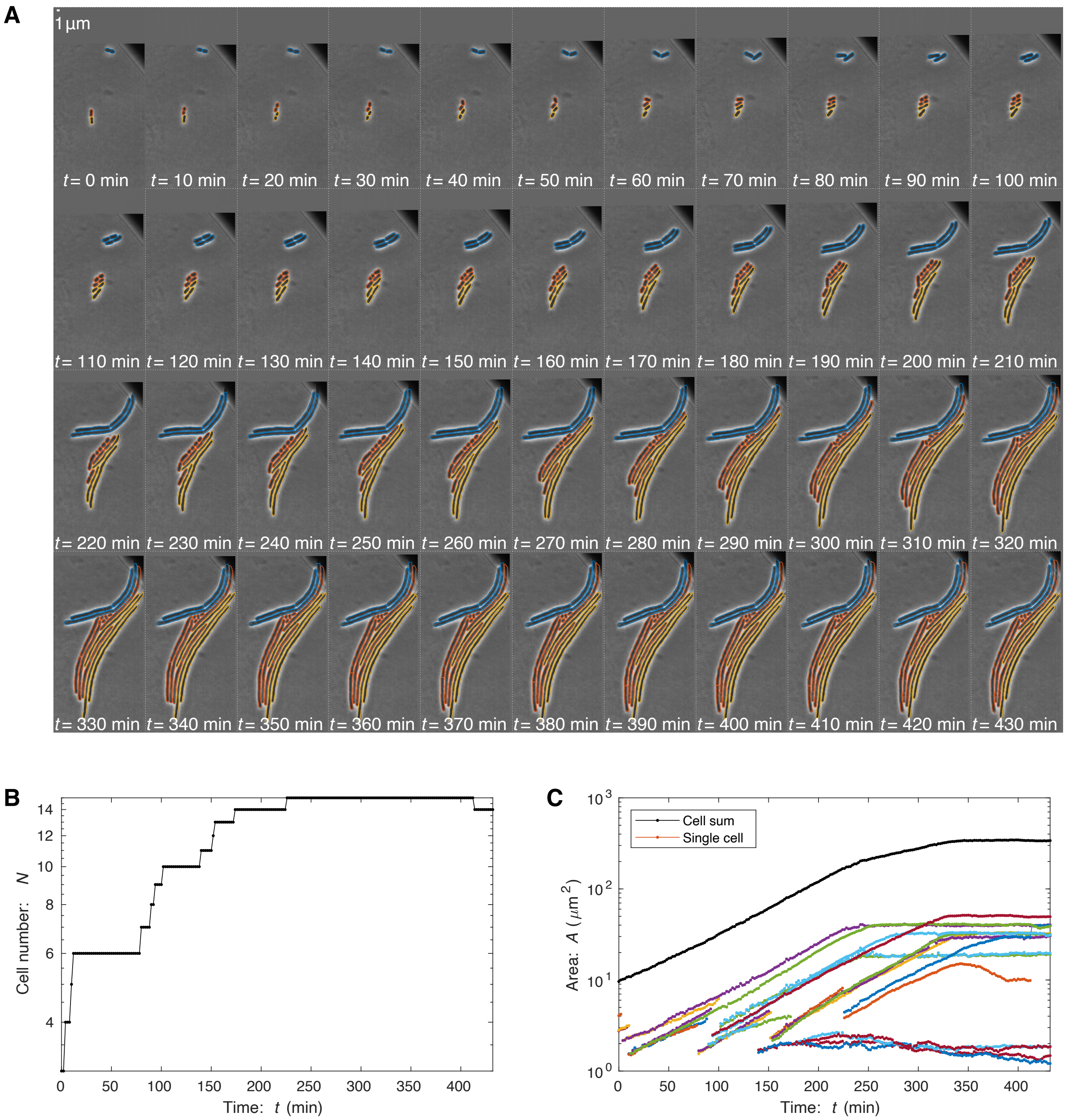

FIG. S6. **Knockout-depletion experiment:** Δ*ftsN*. **Panel A: Frame mosaic.** Three transformants (*ftsN*::*kan*(Km^R), blue, yellow, orange) proliferate. FtsN is an essential cell division protein. Its depletion leads to a failure of the cells to septate. Cells were segmented using OmniSegger for quantitative analysis. **Panel B: Cell number.** The number of transformant progeny as a function of time. After transformation, normal growth persists for roughly 150 min, consistent with FtsN expression being overabundant. **Panel C: Progeny area.** Total progeny-cell area as a function of time. The areal elongation persists even after cell division arrests.

In our statistical analysis, we will initially treat the No-Effect model as the null hypothesis and the Sufficiency model as the alternative hypothesis. If the null hypothesis is rejected, we will then adopt the Sufficiency model as the null hypothesis and adopt the Overabundance model as the alternative hypothesis.

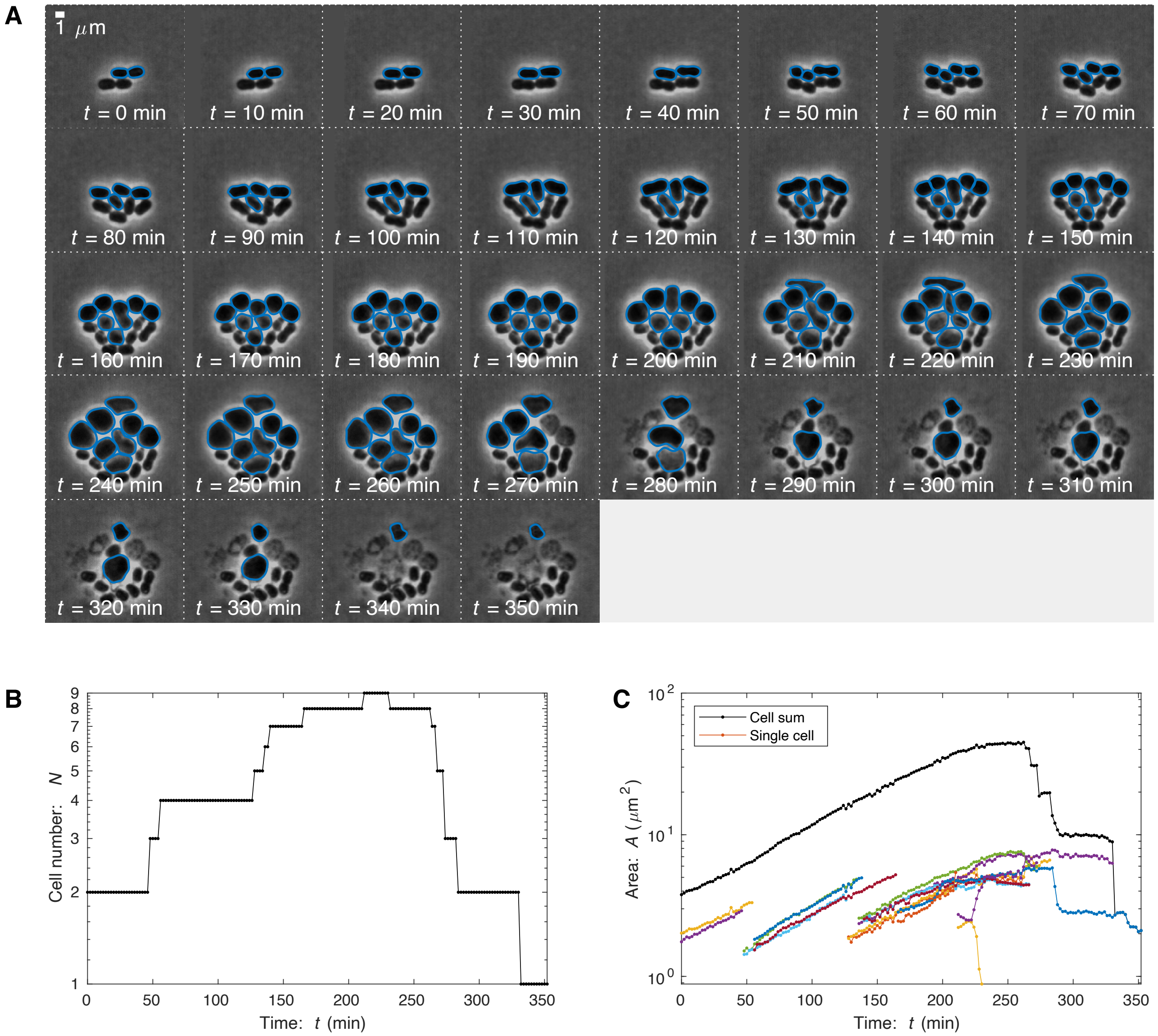

FIG. S7. **Knockout-depletion experiment:** Δ*murA*. **Panel A: Frame mosaic.** Two transformants (*murA*::*kan*(Km^R), blue) proliferate. MurA is an essential enzyme responsible for cell wall precursor synthesis. Its depletion leads to the loss of cell wall integrity, and therefore first the loss of wild-type cell morphology and then cell lysis. Cells were segmented using OmniSegger for quantitative analysis. **Panel B: Cell number.** The number of transformant progeny as a function of time. After transformation, normal growth persists for roughly 200 min, consistent with MurA expression being overabundant. **Panel C: Progeny area.** Total progeny-cell area as a function of time. The areal elongation dynamics are largely consistent with the cell number dynamics: Normal growth persists for roughly 200 min. (This figure shows a more detailed analysis of the data in Fig. 2.)

1. *No-Effect model*

In the *No-effect model*, the mutant has no effect on the growth rate. The abundance in a log culture will therefore be:

$$N_{\rm N}(t;N_0) = N_0 e^{k_0 t}, \tag{S1}$$

where k_0 is the wild-type growth rate and N_0 is the abundance at $t = 0$.

For modeling the TFNseq trajectories, it is the relative abundance that is measured and we therefore normalize by wild-type growth of the culture, resulting in the relative abundance:

$$\eta_{\rm N}(t;\eta_0) = \eta_0, \tag{S2}$$

where η_0 represents the initial relative abundance. (The relative abundance of the No-effect model is independent of t.) Both the abundance $N_{\rm N}$ and relative abundance $\eta_{\rm N}$ are plotted in Fig. S8. Both models depend on

Short name:	Lab number:	Organism:	Genotype:	Source:	Stability:	Selectable marker:	Description:
wild-type	#1139	*A. baylyi*	ADP1	Ref. [43]	Stable	—	Wild-type strain.
YdnaN	#1545	*A. baylyi*	ADP1 *dnaN*::*YPet-dnaN*	This study.	Stable	—	The beta clamp (DnaN) is replaced by the fluorescent fusion *YPet-dnaN* at the endogenous locus.
Δ*IS*	N.A.†	*A. baylyi*	ADP1 *dnaN*::*YPet-dnaN* *ACIA0320-0321*::*kan*	This study.	Stable	Km^R	This is a control strain where non-essential genes, corresponding to an IS element, are knocked out from the YdnaN strain. Even through the strain is stable, it is re-transformed in each knockout-depletion experiment. Transformed strain has a wild-type growth phenotype.
Δ*dnaA*	N.A.†	*A. baylyi*	ADP1 *dnaA*::*kan*	This study.	Unstable	Km^R	DnaA is an essential cell-cycle regulator. This strain must be re-transformed in each knockout-depletion experiment. Transformed strain is wild-type.
Δ*dnaN*	N.A.†	*A. baylyi*	ADP1 *dnaN*::*kan*	This study.	Unstable	Km^R	The beta clamp (DnaN) is an essential component of the replisome. This strain must be re-transformed in each knockout-depletion experiment. Transformed strain is wild-type.
Δ*YdnaN*	N.A.†	*A. baylyi*	ADP1 *YdnaN*::*kan*	This study.	Unstable	Km^R	The beta clamp (DnaN) is an essential component of the replisome. This strain must be re-transformed in each knockout-depletion experiment. Transformed strain is YdnaN (not wild-type).
Δ*murA*	N.A.†	*A. baylyi*	ADP1 *murA*::*kan*	This study.	Unstable	Km^R	The gene product of *murA* is UDP-N-acetylglucosamine 1-carboxyvinyltransferase, an essential protein in synthesizing the precursors of cell wall synthesis. This strain must be re-transformed in each knockout-depletion experiment. Transformed strain is wild-type.
Δ*ftsN*	N.A.†	*A. baylyi*	ADP1 *ftsN*::*kan*	This study.	Unstable	Km^R	The gene product of *ftsN* is essential cell division protein FtsN. This strain must be re-transformed in each knockout-depletion experiment. Transformed strain is wild-type.

TABLE S2. **Summary of strains used in this study.** The *short name* describes the nomenclature of the strains as described in the text. †Strain re-created by transformations in each knockout-depletion experiment are not stable and therefore are not assigned a *lab strain number* and, due to their instability, cannot be distributed.

a single model parameter and are therefore dimension 1.

2. *Sufficiency model*

In the *Sufficiency model*, we model the effect of the mutant as immediate. The cell number is assumed to grow at a new unknown rate:

$$N_{\rm S}(t; N_0, k) = N_0 e^{kt}, \tag{S3}$$

where k is the new growth rate and N_0 is the number of the mutants at $t = 0$. For modeling the TFNseq trajectories, it is the relative abundance that is measured, and we therefore normalize by wild-type growth of the culture, resulting in the relative abundance:

$$\eta_{\rm S}(t; \eta_0, \Delta k) = \eta_0 e^{-\Delta k t}, \tag{S4}$$

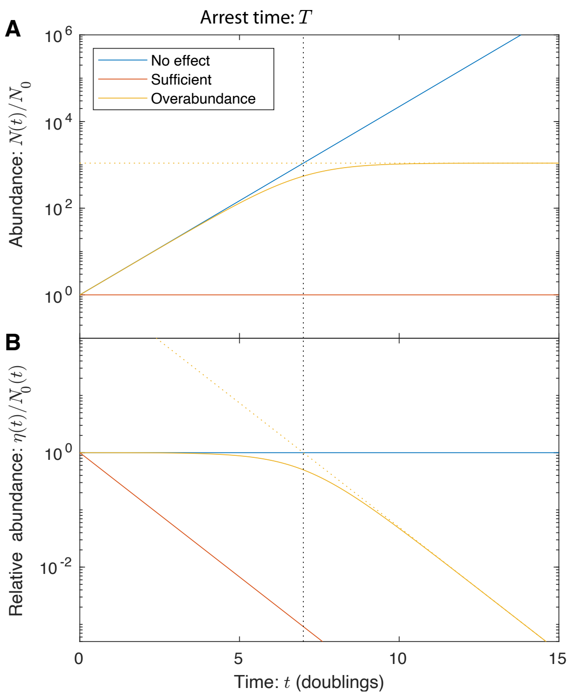

FIG. S8. **Analytic models for growth. Panel A: Mutant abundances for trajectory models.** Mutants described by the *No-effect model* (blue) grow at the wild-type growth rate. Mutants described by the *Sufficient trajectory model* (red) show an immediate change in growth rate after transformation. Mutants described by the *Overabundant trajectory model* (yellow) grow to the arrest time T (black dotted line) with the wild-type growth rate, before adopting a reduced growth rate of $k = 0$. **Panel B: Relative mutant abundances for trajectory models.** Same as above, but abundances are renormalized by wild-type growth.

where $\Delta k \equiv k_0 - k$ is the growth rate reduction of the mutant relative to the wild-type growth rate. Both the abundance N_S and relative abundance η_S are plotted in Fig. S8. Both models depend on two model parameters and are therefore dimension 2. Note that we might naïvely expect $k = 0$ for essential genes; however, we expect some transient growth due to residual protein levels, and these transients will dominate the fit.

3. *Overabundance model*

In the *Overabundance model*, we model the effect of the mutant with a delayed arrest time, T: the transient growth duration as protein dilutes to the threshold level. For short times, the mutant grows with a wild-type rate:

$$N_O = N_0 e^{k_0 t}, \tag{S5}$$

however, at long times we expect growth with a new unknown growth rate k:

$$N_O = N_0' e^{kt}. \tag{S6}$$

We initially attempted to use a piecewise function to join these two limits. However, the sparsity of the data and discontinuous slope at the boundary appeared to give rise to fitting artifacts. In addition, the cell-to-cell variation in protein expression smooths out the transition time. To fix these shortcomings, we adopted an empirical formula with the correct limits, but with a smooth transition at $t = T$:

$$N_O(t; N_0, k, T) = N_0 e^{k_0 t} \frac{e^{\Delta k T} + 1}{e^{\Delta k T} + e^{\Delta k t}}, \tag{S7}$$

where $\Delta k \equiv k_0 - k$ is the loss in growth rate due to the mutation. Modeling the TFNseq trajectories, it is the relative abundance that is measured, and we therefore normalize by wild-type growth of the culture, resulting in the relative abundance:

$$\eta_O(t; \eta_0, k, T) = \eta_0 \frac{e^{\Delta k T} + 1}{e^{\Delta k T} + e^{\Delta k t}}, \tag{S8}$$

where $\Delta k \equiv k_0 - k$ is the growth rate reduction of the mutant relative to the wild-type growth rate. Both the abundance N_O and relative abundance η_O are plotted in Fig. S8. Both models depend on three model parameters and are therefore dimension 3. Note that we might naïvely expect $k = 0$ for essential genes; however, we expect some transient growth due to residual protein levels, and these transients will dominate the fit.

4. *Image processing (cell segmentation) pipeline.*

Cell images were processed using the *OmniSegger* package [47] by running the `processExp` command with default settings. Most of the analysis described in the paper was performed from the `clist.mat` files generated for each dataset.

A. Methods: Cytometry data analyses

Imaging-based analysis for protein overabundance was carried out by assessing the transient cell area growth and septation. The three different single-cell analysis approaches are explained below: *protein abundance*, *area*, and *number analysis*.

1. *Accessing imaging-based cell cytometry data*

Most of the analysis described in the paper was performed using the `clist.mat` files generated for each dataset by the *OmniSegger* package [28, 48, 49]. In particular, the `data3D` field provides time-dependent cell descriptors for each cell in each frame, including `Rod Length`, `Area`, `Fluor1 sum`, and `Fluor1 background`. These descriptors were the input for our analyses. To characterize cell progeny area of fluorescence, we would generate cell lineage trees and cell

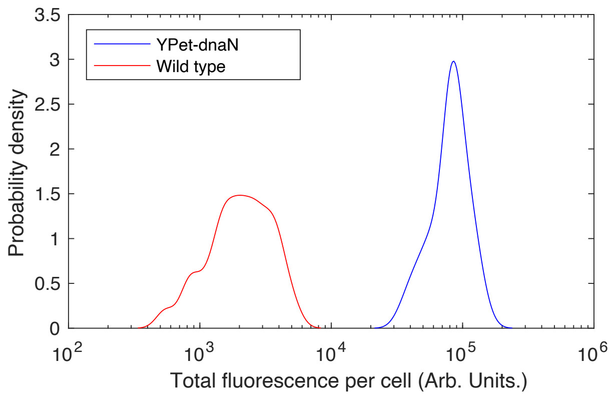

FIG. S9. **Wild-type and *YPet-dnaN* cell can be differentiated by fluorescence intensity with single cell resolution.** Above we characterized fluorescence intensity for wild-type versus *YPet-dnaN* cells. Since the distributions are non-overlapping, the cells can be differentiated by fluorescence microscopy.

progeny IDs using the `getFamily` command and then sum fluorescence or area over all progeny as a function of time. For instance, this data is shown in Fig. S3.

2. *Protein abundance analysis*

To test the hypothesis that the targeted protein is depleted while protein-associated function continues for multiple generations, we visualized YPet-DnaN abundance and localization after the gene was knocked out as described in the paper. In short, we constructed a fluorescent fusion at the endogenous locus to make the YdnaN strain (Materials and Methods), in which the endogenous *dnaN* was replaced by the fusion gene *YPet-dnaN*. In the knockout-depletion experiment, we knocked out the *YPet-dnaN* gene with the *kan* cassette to form *YPet-dnaN*::*kan*.

We determined that wild-type and *YPet-dnaN* cells were unambiguously differentiable by fluorescence intensity. (See Fig. S9.)

To test the protein dilution hypothesis, we measured total progeny fluorescence (the proxy for protein abundance of YPet-DnaN) as a function of time, as the cell progeny proliferated. The dilution model predicts that the protein abundance should scale with the total progeny area like:

$$C(t) = C(0)\frac{A_0}{A(t)}, \qquad \text{(S9)}$$

where $C(t)$ is the protein concentration at time t, C_0 is the abundance at time $t=0$, A_0 is the progenitor area at time $t=0$, and $A(t)$ is the total area of the progeny at time t. In the context of the fusion experiments, the observable is fusion fluorescence, equivalent to an intensity scaling of:

$$I(t) = I(0)\frac{A_0}{A(t)}, \qquad \text{(S10)}$$

where $I(t)$ and $I(0)$ are the average pixel intensity of the progeny at time t and the progenitor at $t=0$. Both area A and intensity I are time-dependent quantities available in the `clist.mat` file. (See Sec. 2 A 1.)

Several successive improvements in the experimental design and analysis were required to test the dilution hypothesis: (i) We initially attempted to image cells at the same frame rate as our phase contrast experiment (1 frame/2 min); however, to resolve YPet-DnaN foci after protein depletion, we had to significantly increase the exposure time of the fluorescence images and decrease the frame rate to avoid phototoxicity and bleaching. Although the predicted scaling (Eq. S10) was immediately observable in the data without corrections at short times, more care was required to observe the depletion at long times. (ii) First, we background subtracted to account for the background fluorescence level, computed as the average intensity in each frame outside the cell masks. This correction significantly improved the agreement with Eq. S10 at intermediate times, but did not yet account for cellular autofluorescence. (iii) Next, we analyzed a mixture of wild-type and *YdnaN* cells, using the intensity of the wild-type cell in the same microcolony for the background subtraction. This method led to good agreement with Eq. S10 even at long times (Fig. 1).

Why was a mixture of wild-type and *YdnaN* cells preferable to imaging the two strains independently? A detailed analysis of single cell intensities revealed that wild-type cells in close proximity to *YdnaN* cells in the microcolony had higher pixel intensity, due to the diffuse halo created by the bright *YdnaN* cells. The use of wild-type cells in the same field of view helped correct for the diffuse fluorescent light necessary for the analysis of protein abundance at large depletion times. Cell fluorescence intensities at $t=0$ are used to differentiate between wild-type and *YdnaN* cells.

3. *Areal growth analysis*

In this section, we develop the statistical model for the analysis of cell-area based growth assays to determine both the model parameters and the statistical uncertainty of parameters on a per-experiment basis. We provide this development for completeness; **however, cell-to-cell variation will dominate the reported errors**.

Statistical procedure. For the imaging-based analyses, we define the following statistical procedure: For the analysis of essential genes, we will fix the asymptotic growth rate $k=0$. Therefore, the Sufficiency model is now considered the null hypothesis since it is the lowest dimensional model. The first alternative hypothesis is the No-effect model, where the wild-type growth rate k_0 is fit in each analysis. If the Sufficiency model is rejected, we then adopt the No-effect model as the new null hypothesis and adopt the Overabundance model as the new alternative hypothesis.

Areal growth models. This growth metric is sensitive to cell elongation (rather than septation). Let $A(t)$ be the observed area of all cells sharing a single progenitor cell. For the areal growth model, we substitute cell area $A(t)$ for the abundance $N(t)$ and A_0 for N_0 in Eqs. S1, S3, and S7. The models are:

$$\ln A_{\rm S}(t;A_0) = \ln A_0, \quad \text{(S11)}$$
$$\ln A_{\rm N}(t;A_0,k_0) = \ln A_0 + k_0 t, \quad \text{(S12)}$$
$$\ln A_{\rm O}(t;A_0,k_0,T) = \ln A_0 + k_0 t + \ln \tfrac{e^{k_0 T}+1}{e^{k_0 T}+e^{k_0 t}}, \quad \text{(S13)}$$

where we have substituted $k = 0$.

Statistical model for areal growth analysis. We will model the error associated with determining the area of the cells as proportional to cell number or area:

$$\sigma_A \propto A(t). \quad \text{(S14)}$$

This model is consistent with many mechanisms. Rather than fitting a model with a variable error, it is more convenient to introduce a new variable, a, with constant error:

$$a(t) \equiv \ln A(t). \quad \text{(S15)}$$

Since $\mathrm{d}a = \mathrm{d}A/A$, then $\sigma_a = \sigma_A/A$ which leads to an analysis with constant error.

The Shannon Information (minus log likelihood) for the log area in frame i is:

$$h(a_i|\boldsymbol{\theta}) = \tfrac{1}{2}\ln 2\pi\sigma_a^2 + \tfrac{1}{2\sigma_a^2}[a_i - \mu_a(t_i;\boldsymbol{\theta})]^2, \quad \text{(S16)}$$

where $\boldsymbol{\theta}$ represents the parameter vector, μ_a is the time-dependent mean log area defined by the growth models (Eqs. S11-S13). For a time series with $i = 1...N$ frames, the total Shannon information is:

$$h(\{a_{i=1...N}\}|\boldsymbol{\theta}) = \tfrac{N}{2}\ln 2\pi\sigma_a^2 + \tfrac{1}{2\sigma_a^2}S^2, \quad \text{(S17)}$$

which can be formulated as a least-squares minimization where:

$$\Delta a_i \equiv a_i - \mu_a(t_i;\boldsymbol{\theta}), \quad \text{(S18)}$$
$$S^2(\boldsymbol{\theta}) = \sum_{i=1}^{N} \Delta a_i^2, \quad \text{(S19)}$$

where i is the frame index.

Estimate of error for areal growth analysis. We will statistically estimate the relative area uncertainty (σ_a) from the wild-type growth data. The expression for the MLE for σ_a^2 is:

$$\hat{\sigma}^2_{a,\rm MLE} = \tfrac{1}{N} S^2(\hat{k}_0, \hat{a}_0), \quad \text{(S20)}$$

where Eq. S19 is evaluated at the MLE values of the other parameters for the No-effect model. There is one additional improvement to this estimate which is straight forward to implement. It is well known that Eq. S20 is biased from below. We can construct an unbiased estimator by correcting for the complexity of the model for the mean (dimension two) [46]:

$$\hat{\sigma}_a^2 = \tfrac{1}{N-2} S^2(\hat{k}_0, \hat{a}_0), \quad \text{(S21)}$$

which we will use for our variance estimator. Note that if only a single mean were fit, the prefactor would be $(N-1)^{-1}$ accounting for the one model dimension; however, since we fit both the slope and the offset, the prefactor is $(N-2)^{-1}$ accounting for the two model dimensions [46, 50].

From the wild-type growth data, the unbiased estimator for the error for log area (Eq. S21) is:

$$\sigma_a = 1.5 \times 10^{-3}, \quad \text{(S22)}$$

or alternatively, this result can be stated in a more intuitive form: There is a 0.15% error in the cell area.

Application to observed data. To determine the model parameters, we will minimize the Shannon information (Eq. S34) numerically, by a least-squares minimization of Eq. S18. We estimate the Fisher information using the resulting Jacobian from the least-squares minimization:

$$\hat{I} \equiv \tfrac{1}{\sigma_a^2} J J^T, \quad \text{(S23)}$$

where the Jacobian matrices J are contracted over the frame index and σ_a is given by Eq. S22. The parameter uncertainties are then estimated from the Fisher information (Eq. S76). Although Eq. S76 accounts for the statistical uncertainty in the parameters, it does not account for the cell-to-cell variation in protein abundance. We found that this cell-to-cell variation was dominant. We therefore cite this cell-to-cell variation-based uncertainty. For the p-value calculations (Eq. S79), we compute the test statistic λ (Eq. S77) from the differences between residual norms for the null and alternative hypotheses:

$$\lambda = \tfrac{1}{2\sigma_a^2}(S_0^2 - S_1^2), \quad \text{(S24)}$$

where σ_a is given by Eq. S22, and the residual norms for model I (the null (0) or the alternative (1) hypotheses) are defined in Eq. S19.

B. Methods: Cell-number growth analysis

In this section, we develop the statistical model for the analysis of cell-number based growth assays to determine both the model parameters and the statistical uncertainty of parameters on a per-experiment basis. We provide this development for completeness; **however, cell-to-cell variation will dominate the reported errors**.

Statistical procedure. For the imaging-based analyses, we define the following statistical procedure: For the analysis of essential genes, we will fix the asymptotic growth rate $k = 0$. Therefore, the Sufficiency model is now considered the null hypothesis since it is the lowest dimensional model. The first alternative hypothesis is the No-effect model where the wild-type growth rate k_0 is fit in each analysis. If the Sufficiency model is rejected, we then adopt the No-effect model as the new null hypothesis and adopt the Overabundance model as the new alternative hypothesis.

Cell-number growth models. For the cell-number growth model, we use Eqs. S1, S3, and S7. The statistical models depend on the growth rates as function of time for model I, which we define as:

$$k_I = \tfrac{\partial}{\partial t} \ln N_I(t;\theta_I), \tag{S25}$$

where N_I is the cell abundance in model I at time t. The growth rates for the respective models are:

$$k_N(t;k_0) = k_0, \tag{S26}$$

$$k_S(t) = 0, \tag{S27}$$

$$k_O(t;k_0,T) = k_0 \cdot [1+e^{k_0(t-T)}]^{-1}, \tag{S28}$$

where Eq. S28 interpolates between the initial growth rate k_0 and final growth rate $k = 0$ at time T.

Deriving the Shannon information. Consider an experiment in which images are taken with a high frame rate, where the time duration between frames is δt. Let the frame number be denoted $I = 1...m$ and the number of cells in each frame N_I. Let the model for cell growth be formulated such that the growth rate at time t_I is:

$$k_I = k(t_I;\theta), \tag{S29}$$

where θ represents a parameter vector. In this analysis, we model cell division as a Markovian process where:

$$\dot{N} = kN, \tag{S30}$$

which is to say that we will ignore the internal state of cells. For instance, at time t, cells have the same rate of division, irrespective of cell age.

In this model, the number of cell divisions n_I that occur over the short time interval δt is Poisson distributed:

$$q(n_I|\mu_I) = \tfrac{\mu_I^{n_I}}{n_I!}e^{-\mu_I}, \tag{S31}$$

where

$$\mu_I \equiv \delta t N_I k(t_I;\theta), \tag{S32}$$

is the mean number of divisions.

We now compute the Shannon information associated with the entire experiment:

$$h(\{N_I\}_{I=1...m}|\theta) = -\sum_{I=1}^{m} \ln q(n_I|\mu_I). \tag{S33}$$

Substituting Eqs. S31 and S32, the equation is simplified to:

$$h = \sum_{I=1}^{m} \delta t N_I k_I - \sum_{I\in \mathrm{Div}} n_I \ln \delta t N_I k_I + \sum_{I\in \mathrm{Div}} \ln n_I!, \tag{S34}$$

where Div represents the frames immediately preceding division. For instance, if there is one cell at frame 5 and two cells at frame 6, Div $= \{5\}$.

Application to observed data. To determine the model parameters (Eq. S75), we will minimize the Shannon information (Eq. S34) numerically, and determine the Hessian at the optimal parameter values to estimate the Fisher information:

$$\hat{I}_{ij} = H_{ij}, \tag{S35}$$

where H is the Hessian matrix. The parameter uncertainties are then estimated from the Fisher information (Eq. S76). We therefore cite this cell-to-cell variation-based uncertainty. For the p-value calculations (Eq. S79), we compute the test statistic λ (Eq. S77) from the differences in the Shannon information (Eq. S34).

Error estimates of arrest times in imaging-based approach. Due to the noisiness of gene expression, there is significant cell-to-cell variation in protein abundance from progenitor to progenitor in the image-based analysis; however, there is significantly less noise in protein partitioning between the daughter cells [24]. Eq. S76 accounts for the statistical uncertainty in the parameters for a single progenitor cell, but it does not capture the uncertainty due to cell-to-cell variation. To estimate the uncertainty associated with cell-to-cell variation, we used the canonical unbiased empirical error estimator [46]:

$$\hat{\sigma}^2_{\hat{\theta}} \equiv \tfrac{1}{N-1}\sum_{i=1}^{N}(\hat{\mu}_i - \overline{\hat{\mu}})^2, \tag{S36}$$

$$\overline{\hat{\mu}} \equiv \tfrac{1}{N}\sum_{i=1}^{N}\hat{\mu}_i, \tag{S37}$$

where index i runs over the progenitor cells $1..N$ and $\hat{\mu}_i$ is the estimate of the parameters from progenitor i. We found that this cell-to-cell variation (Eq. S36) was larger than the per-progenitor estimated error (Eq. S76).

3. STATISTICAL ANALYSIS OF TFNSEQ TRAJECTORIES

A. Methods: Time correction

Since the mutants transition from lag phase to log phase after transformation, we used a log phase equivalent time for the TFNseq-approach analysis. The corrected sampling times (t_s) are estimated from the num-

ber of doublings (D_s) for the non-essential mutants obtained from TFNseq experiment([18]):

$$t_s = D_s * [doubling\ time]. \tag{S38}$$

For our experiment, the doubling time for ADP1 in M9 at 30°C is 37 min.

B. Methods: Defining the likelihood

We assume that deep-sequencing is well modeled by a Poisson process for which the probability mass function is:

$$p(n|\mu) = \frac{\mu^n}{n!}e^{-\mu}, \tag{S39}$$

where n is the number of reads and μ is the mean-number parameter. For large n, we use the normal-distribution approximation:

$$p(n|\mu) \approx \frac{1}{\sqrt{2\pi\mu}} \exp\left[-\frac{(n-\mu)^2}{2\mu}\right]. \tag{S40}$$

The total likelihood for sequential observations $n_{1...m}$ at time $t_{1...m}$ is therefore:

$$q(n_{1...m}|\theta_I) = \prod_{i=1}^{m} p(n_i|\mu)|_{\mu=N_I(t_i|\theta_I)}, \tag{S41}$$

where N_I is one of the trajectory models and θ_I is the parameter vector for model I. The Shannon information is:

$$h(n_{1...m}|\theta_I) \equiv -\ln q(n_{1...m}|\theta_I), \tag{S42}$$

$$= -\sum_{i=1}^{m} \ln p(n_i|\mu)|_{\mu=N_I(t_i|\theta_I)}. \tag{S43}$$

To fit this expression, we use the total number of reads mapped to each mutant gene as the n_i and minimize to determine the MLE parameter estimates.

C. Methods: Data binning for overabundance plot

We used two different binning approaches to show the experimental trend in overabundance (o) as a function of transcription (μ_m).

1. μ-binning

For the μ-binning approach, we used the canonical approach: We divided the log-10 interval of μ_m into bins of width $\Delta\log_{10}\mu_m = 0.5$. The resulting binning is shown in Fig. S12A.

The shortcoming with this approach is that there is uncertainty in both x and y axes (transcription measurements (message number μ_m) and overabundance (o) respectively) and there is a steep slope of the predicted message number as $\mu_m \to 1$ which obscures the trend for the data at low transcription levels.

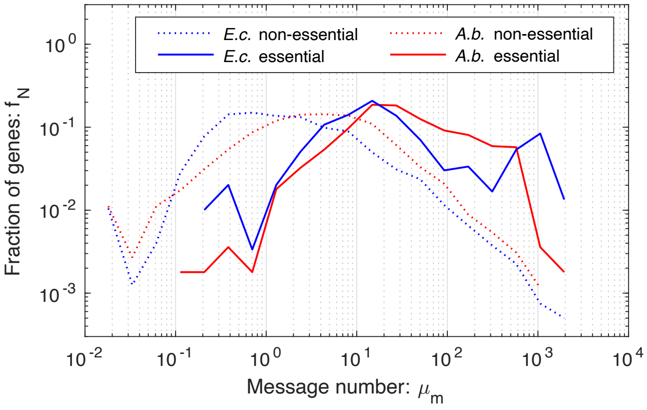

FIG. S10. **Message number distributions for essential and non-essential genes in *E. coli* and *A. baylyi*.** Nearly all *A. baylyi* essential genes are expressed above the one-message-per-cell-cycle threshold. This distribution of both non-essential and essential genes in *A. baylyi* is qualitatively similar to that in *E. coli*, as predicted [11].

2. n-binning

To better capture the data trend as $\mu_m \to 1$, we binned the data using an alternative dependent variable, n_m. The threshold transcription level n_m is defined as:

$$n_m \equiv \mu_m/o, \tag{S44}$$

and can be understood as the transcription level that would be required in RLTO model, in the absence of gene-expression noise, for growth. We used an identical binning for n_m: $\Delta\log_{10} n_m = 0.5$. The resulting binned is shown in Fig. S12B.

D. Results: Overabundance is consistent between two knockout-depletion replicate experiments.

Two independent knockout-depletion experiments were performed [18]. Qualitatively, the data from Experiment 1 fit the kinetic model better and therefore we used this dataset for our primary analysis. However, the entire analysis was repeated for Experiment 2 data and the results are qualitatively unchanged. The overabundances for both experiments are reported in the Supplementary Data S1. The inferred replicate overabundance values are compared in Fig. S13.

We used the second dataset to estimate experimental uncertainties in the reported log overabundance values by estimating the variance from the two observed data points for each gene i:

$$\sigma^2_{\log_{10} o,i} = \tfrac{1}{2}[\log_{10} o_i(\text{Exp 1}) - \log_{10} o_i(\text{Exp 2})]^2. \tag{S45}$$

To quantify the typical experiment-to-experiment variation, we computed the gene-averaged variance in the

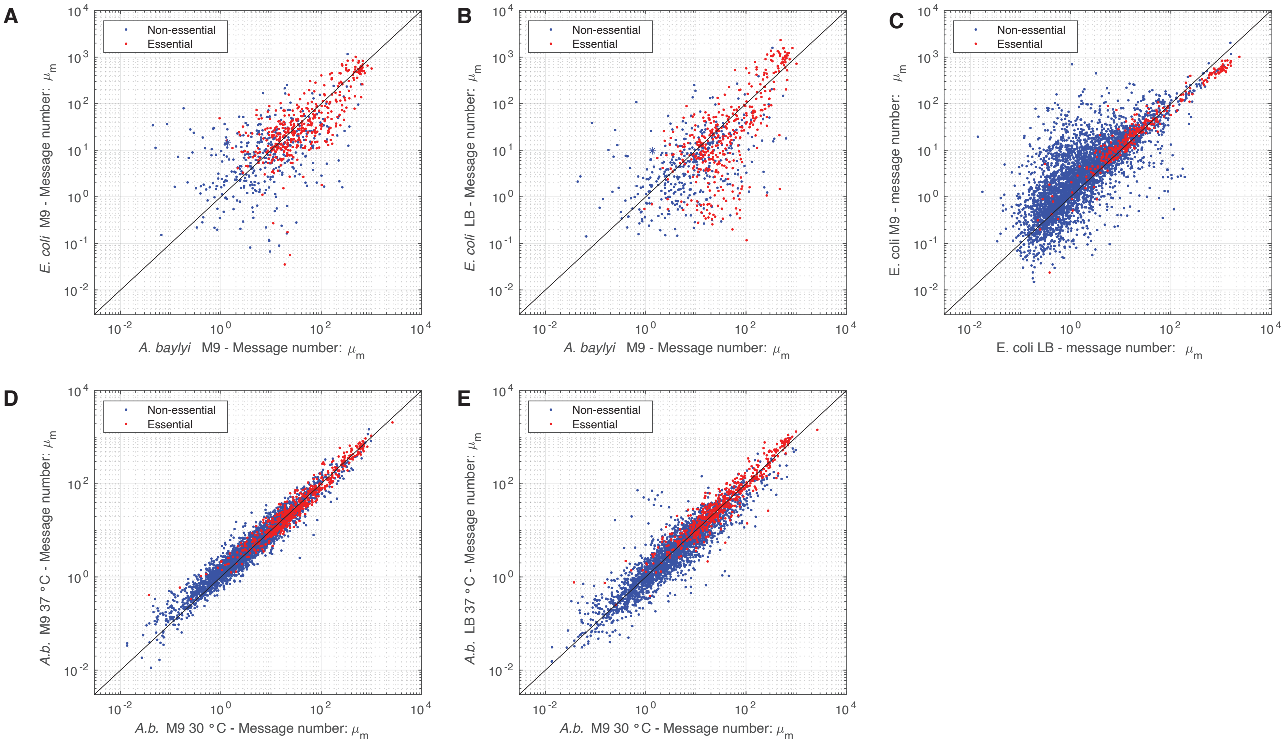

FIG. S11. **Transcriptome comparisons. Panel A: *E. coli* on M9 versus *A. baylyi* on M9. Panel B: *E. coli* on LB versus *A. baylyi* on M9. Panel C: *E. coli* on M9 versus on LB. Panel D: *A. baylyi* on M9 at 37°C versus at 30°C. Panel E: *A. baylyi* on LB at 37°C versus on M9 at 30°C .** Throughout, there is broad consistency between the expression levels (message number) of genes, both between organisms and between conditions. These observations suggest a consistent overall transcriptional program governs gene expression both between organisms and growth conditions.

log overabundance over essential genes. The variance and standard deviation are:

$$\sigma^2_{\log_{10} o} = 0.17, \quad \text{(S46)}$$

$$\sigma_{\log_{10} o} = 0.42, \quad \text{(S47)}$$

which corresponds to a multiplicative uncertainty of roughly 2.6-fold in the overabundance. We conclude that the inferred overabundance is broadly consistent between the experiments.

E. Methods: Detection limit

We define the detection limit λ_{min} for the TFNseq experiments as the Poisson rate at which the mutant would be detected 95% of the time. This corresponds to the probability of non-detection (i.e. not measuring a count) as 5%. Therefore:

$$0.05 = p_0(\lambda_{\text{min}}) = \exp(-\lambda_{\text{min}}), \quad \text{(S48)}$$

where p_0 is the Poisson probability for zero counts. The detection limit is therefore:

$$\lambda_{\text{min}} = 3.0 \text{ reads}. \quad \text{(S49)}$$

Class	Terms
GO:0006260	DNA replication
GO:0051301	Cell division
GO:0008610	Lipid biosynthetic process
GO:0009252	Peptidoglycan biosynthetic process
GO:0008643	Carbohydrate transport
GO:0006355	Regulation of DNA-templated transcription
GO:0003824	Catalytic activity
GO:0003677	DNA binding

TABLE S3. **Gene ontology classifications and terms.** A summary of the gene ontology classifications and terms used in the study.

F. Methods: Analysis of overabundance for different Gene Ontologies (GO)

To classify genes, the gene ontology classifications and terms summarized in Tab. S3 were used.

We analyzed only genes in *A. baylyi* that had homologues in *E. coli*. The *E. coli* classifications were downloaded from EcoCyc database [51].

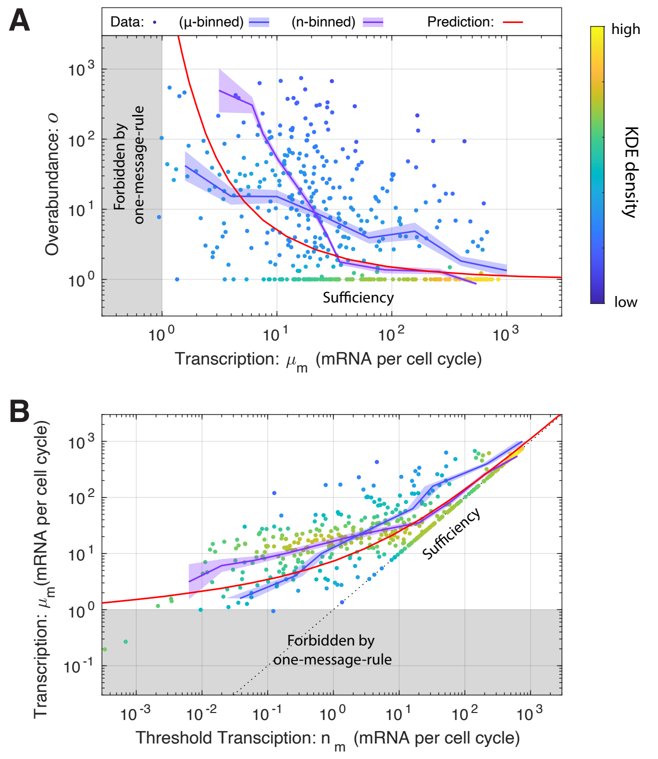

FIG. S12. **Low expression proteins have high overabundance. Panel A: Overabundance versus transcription.** The measured transcription-overabundance pairs are shown for essential genes (including estimated gene density.) The blue curve shows the μ-binned data. The purple curve shows the n-binned data. The RLTO model (red) predicts that overabundance grows rapidly as the transcription level is reduced. **Panel B: Transcription versus threshold transcription.** Alternatively, the message number μ_m is shown as a function of the inferred threshold message number: $n_m \equiv \mu_m/o$. The blue curve shows the μ-binned data. The purple curve shows the n-binned data.

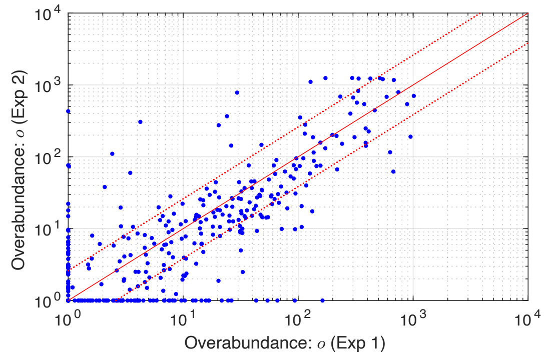

FIG. S13. **Overabundance is consistent between two knockout-depletion replicate experiments.** The inferred overabundance values are compared for two experimental replicates. Perfectly matched experiments would align on the diagonal (red). The inferred overabundance values are broadly consistent between the two experiments.

G. Analysis of overabundance for different gene regulatory controls

To investigate the effect of transcriptional regulation in determining protein overabundance, we assumed that the regulatory network in *A. baylyi* is roughly equivalent to that in *E. coli* which has been much more extensively studied. We used the EcoCyc database [51] to generate a list for each gene i of the list of direct regulators. For each gene, we counted the direct regulators of each gene, then ranked the genes in terms of regulator number, and finally we defined the top 10% of the genes as *highly regulated*. We also generated a list of genes directly inhibited by each gene. If a gene directly inhibited itself, we defined the gene as *autoregulatory*.

4. RNA-SEQ ANALYSIS OF TRANSCRIPTION

A. Methods: RNA-Seq protocol

1. *RNA extraction*

ADP1 RNA was harvested using methods developed by Culviner et al. [52]. Total RNA was harvested by mixing 1ml of *A. baylyi* (≈0.5 OD) with 110ul of ice-cold stop solution (95% ethanol and 5% acid-buffered phenol) and spinning in a tabletop centrifuge for 30 s at 13,000 rpm. The supernatant was flash-frozen and stored at -80°C until RNA extraction is ready. To start RNA extraction, 1ml of heated 65°C was added to the sample. The mixture was shaken at 65°C for 10 min and flash-frozen at -80°C for at least 10 min. The pellets were thawed at room temperature and spun at top speed in a benchtop centrifuge at 4°C for 5 min. The supernatant was collected and added to 400 µl of 100% ethanol. The mixture was passed through DirectZol spin column (Zymo). The column was washed twice with RNA prewash buffer and once with RNA wash buffer (Zymo). RNA was eluted from the column with 90 µl diethyl pyrocarbonate (DEPC)-H2O. Genomic DNA was removed with 4 µl of Turbo DNase I (Invitrogen) and supplemented with 10µl of 10x Turbo DNase I buffer to a final volume of 100µl. The solution was heated to 37°C for 40 min. Then RNA was diluted with 100 µl DEPC-H2O, extracted with 200 µl buffered acid phenol-chloroform, followed by ethanol precipitation at -80°C for 4 h with 20 µl of 3 M sodium acetate (NaOAc), 2 µl GlycoBlue (Invitrogen), and 600 µl ice-cold ethanol. To pellet RNA, the samples were centrifuged at 4°C for 30 min at 21,000 × g. The pellets were washed twice with 500 µl of ice-cold 70% ethanol, followed by centrifugation at 4°C for 5 min. RNA pellets were then air dried and resuspended in 50 µl DEPC-H2O. The yield and integrity of RNA was verified with NanoDrop spectrophotometer, and by running 50 ng of total RNA on a Novex 6% Tris-buffered EDTA (TBE)-urea polyacrylamide gel (Invitrogen).

2. *rRNA Depletion*

rRNA was depleted through the DIY method developed by Culviner *et al.* [52] as well. We used their 21 biotinylated oligonucleotides for *E. coli*. The selected biotinylated oligonucleotides were synthesized by IDT and resuspended to 100 µM in TE buffer (Qiagen). An oligonucleotide mixture was made by mixing equal volumes of each 16S and 23S primers and double volumes of 5S primers. The pooled mixture was diluted with DEPC treated H2O based on the total RNA, using their Excel-based calculator. Using the Excel-based calculator, the calculated volume of Dynabeads MyOne streptavidin C1 beads (ThermoFisher) were washed three times in equal volume of 1x B&W buffer, resuspended in 30 µl of 2× B&W buffer and supplemented with 1µl of SUPERase-In RNase inhibitor (ThermoFisher). The beads were set aside at room temperature until the probes were ready to be pulled down. To collect rRNA, 2 to 3µg total RNA and 1 µl of the diluted biotinylated probe mix were combined on ice into a final annealing reaction mixture of 1xSSC and 500 µM EDTA. All the appropriate volumes were computed using the Excel-based calculator. The RNA and probe mixture was incubated at 70°C for 5 min, and slowly cooled to 25°C at a rate of 1°C per 30 s. The annealed mixture was then added to 30 µl of beads that were resuspended in 2× B&W buffer. The mixture was mixed by pipetting and vortexing at medium speed, and followed by incubating for 5 min at room temperature. The reaction mixtures were then vortexed, and incubated at 50°C for 5 min. To pull down the biotinylated probes, the reaction mixtures were placed immediately placed on the magnetic rack. The supernatant was carefully pipetted, placed on ice, and diluted to 200 µl in DEPC-H2O. The RNA was purified through ethanol precipitation with 20 µl of 3 M NaOAc, 2 µl GlycoBlue (Invitrogen), and 600 µl ice-cold ethanol at -20°C for at least 1 h. To pellet RNA, the samples were centrifuged at 4°C for 30 min at 21,000 × g. The pellets were washed twice with 500 µl of ice-cold 70% ethanol, followed by centrifugation at 4°C for 5 min. RNA pellets were then air dried and resuspended in 10 µl DEPC-H2O. The yield and rRNA depletion effectiveness were verified with NanoDrop spectrophotometer, and by running 50 ng of total RNA on a Novex 6% Tris-buffered EDTA (TBE)-urea polyacrylamide gel (Invitrogen). The yield and integrity of the library was checked by running the samples in qPCR using NEBNext Library Quant Kit for Illumina(NEB) and the Bioanalyzer.

3. *Library prep and sequencing*

The RNA library was prepared with NEBNext® Multiplex Oligos for Illumina(NEB) and NEBNext ultra II RNA Library Prep Kit for Illumina(NEB). For the library prep protocol, we followed section 4 of the kit's provided protocol: Protocols for use with Purified mRNA or rRNA Depleted RNA. The quality of the final library was verified by running the samples on high sensitivity Bioanalyzer chip. The samples were pooled to a final concentration of 8.5nM, and were sequenced with NextSeq 150 cycle kit.

B. Methods: Computation of message number

To estimate the message number for gene i, defined as the total number of mRNA molecules transcribed per cell cycle, from the RNA-Seq data, we use the approach we described earlier [11]. Let the relative number of reads for gene i be r_i:

$$r_i = \frac{N_i}{N_{\rm tot}}, \tag{S50}$$

where N_i is the reads per kilobase (rpk) for gene i and $N_{\rm tot}$ is the rpk for all genes. We apply two different re-scalings: First, we re-scale the relative message abundance to reflect the cellular abundance of the message, and then we scale this number by the ratio of cell cycle duration to mRNA lifetime to estimate the number of times a gene is transcribed per cell cycle. For *A. baylyi*, we use the same scaling factor as *E. coli*:

$$\mu_{m,i} = 9.4 \times 10^4 \cdot r_i, \tag{S51}$$

where $\mu_{m,i}$ is the estimated message number (number of mRNA molecules transcribed per cell cycle).

To check the consistency of this estimate, we generated histograms for message number for essential and non-essential genes, and compared them to the histograms for *E. coli*. We expect the distribution of essential message numbers to abut 1 message per cell cycle, while non-essential genes can be expressed at significantly lower levels. The observed distribution is consistent with this expectation. (See Fig. S10.)

C. Results: Comparison of *A. baylyi* and *E. coli* gene expression

Knockout-depletion experiments are not tractable in *E. coli* and many other model systems. It is therefore difficult to directly test the overabundance hypothesis in these other systems. However, it is possible to determine if *E. coli* expression patterns are consistent with overabundance.

If overabundance were specific to *A. baylyi*, we would expect to see higher relative transcription of lower abundance essential genes in *A. baylyi*, where overabundance is large, relative to *E. coli* if its expression levels were sufficient. Fig. S11 compares the message number between homologues in the two organisms and between growth conditions within a particular organism for all genes.

In short, we find that the transcription levels of homologous genes are broadly consistent between *A. baylyi* and *E. coli*. In other words, there is no evidence that

A. baylyi elevates the expression of low expression genes relative to *E. coli*. This null result is consistent with the model that gene expression programs are consistent between the two organisms (*i.e.* both use overabundance).

5. ROBUSTNESS LOAD TRADE-OFF (RLTO) MODEL

We have provided a detailed description of the Robustness Load Trade-Off (RLTO) Model in Ref. [11]; however, in the interest of making this paper self-contained, we provide a concise summary of key elements and results from that paper in this supplementary section.

A. Methods: Detailed description of the noise model

1. *Stochastic kinetic model for the central dogma.*

The canonical steady-state noise model for the central dogma describes multiple steps in the gene expression process [35, 36, 53]: Transcription generates mRNA messages. These messages are then translated to synthesize the protein gene products [54]. Both mRNA and protein are subject to degradation and dilution [55]. At the single cell level, each of these processes are stochastic. We will model these processes with the stochastic kinetic scheme [54]:

$$\begin{array}{ccccc} \text{DNA} & \xrightarrow{\beta_m} & \text{mRNA} & \xrightarrow{\beta_p} & \text{Protein} \\ & & \gamma_m \big\downarrow & & \gamma_p \big\downarrow \\ & & \varnothing & & \varnothing, \end{array} \tag{S52}$$

where β_m is the transcription rate (s^{-1}), β_p is the translation rate (s^{-1}), γ_m is the message degradation rate (s^{-1}), and γ_p is the protein effective degradation rate (s^{-1}). The message lifetime is $T_m \equiv \gamma_m^{-1}$. For most proteins in the context of rapid growth, dilution is the dominant mechanism of protein depletion and therefore γ_p is approximately the growth rate [53, 56, 57]: $\gamma_p = T^{-1}\ln 2$, where T is the doubling time.

2. *Statistical model for single-cell protein abundance.*

Consistent with previous reports [35, 36], we find that the distribution of protein number per cell (at cell birth) was described by a gamma distribution [11]:

$$N_p \sim \Gamma(a, \theta), \tag{S53}$$

where N_p is the protein number at cell birth and Γ is the gamma distribution, which is parameterized by a scale parameter θ and a shape parameter a. (See Sec. 7 A.) We refer to this distribution as the *canonical steady-state noise model*; The relation between the four kinetic parameters and these two statistical parameters has already been reported, and have clear biological interpretations [36]: The scale parameter:

$$\theta = \varepsilon \ln 2, \tag{S54}$$

is proportional to the translation efficiency:

$$\varepsilon \equiv \tfrac{\beta_p}{\gamma_m}, \tag{S55}$$

where β_p is the translation rate and γ_m is the message degradation rate. ε is understood as the mean number of proteins translated from each message transcribed. The shape parameter a can also be expressed in terms of the kinetic parameters [36]:

$$a = \tfrac{\beta_m}{\gamma_p}; \tag{S56}$$

however, we will find it more convenient to express the scale parameter in terms of the cell-cycle message number:

$$\mu_m \equiv \beta_m T = a \ln 2, \tag{S57}$$

which can be interpreted as the mean number of messages transcribed per cell cycle. Henceforth, we will abbreviate this quantity *message number* in the interest of brevity.

B. Methods: Summary of the RLTO fitness model

1. *Metabolic load in the RLTO model*

To produce a minimal model to study the trade-off between robustness and metabolic load, we must consider both the metabolic cost of transcription and translation. We will write that the metabolic load (in protein equivalents) associated with gene i is:

$$\delta N_i = \lambda \mu_{m,i} + \mu_{p,i}, \tag{S58}$$

where λ is the message cost, the metabolic load associated with an mRNA molecule relative to a single protein molecule of the gene product.

$$\ln \tfrac{k}{k_0} = -\tfrac{(\lambda+\varepsilon_i)\mu_{m,i}}{N_0}. \tag{S59}$$

This equation has an intuitive interpretation: growth slows in proportion to the relative added metabolic load. In resource allocation models [58], the capacity of the cell for growth can increase as protein sectors increase in size. In our context, this does not occur since we consider the uncoordinated changes in the levels of single proteins. *I.e.* we assume some other protein of factor is rate limiting. See the detailed discussion in Ref. [11].

2. *Growth rate with stochastic arrest*

As discussed in Ref. [11], we idealize the slow growth associated with essential proteins falling below threshold as growth arrest. This arrest model has phenomenology consistent with more detailed and realistic models where cells experience a significant growth slowdown rather than true growth arrest [11].

In the idealized growth arrest model, if all essential proteins are above threshold, the cell cycle duration τ is determined by the metabolic load predictions (Eq. S59); however, if any essential protein is below threshold, the cell cycle duration is infinite. The probability mass function for the cycle-cycle duration T interpreted as a random variable is therefore:

$$p_T(t) = \begin{cases} P_+, & t = \tau \\ (1 - P_+), & t \to \infty \end{cases}. \tag{S60}$$

As we show in Ref. [11], the growth rate can be computed exactly:

$$k = \tau^{-1} \ln(2P_+). \tag{S61}$$

Note that Eq. S61 is equivalent to the *Euler-Lotka* equation [59, 60]. As expected, the growth rate goes down as the probability of growth P_+ decreases, stopping completely at $P_+ = \frac{1}{2}$. We can then compute the ratio of the growth with (k) and without arrest (k_0):

$$\ln \tfrac{k}{k_0} = \tfrac{1}{\ln 2} \ln P_+, \tag{S62}$$

where k_0 is computed by evaluating Eq. S61 at $P_+ = 1$ and we have assumed the difference between k and k_0 is small.

3. *RLTO growth rate*

In the RLTO model, we will assume the probability of growth is the probability that all essential protein numbers are above threshold. We will further assume that each protein number is independent, and therefore:

$$P_+ = \prod_{i \in \mathscr{E}} \Pr\{N_{p,i} > n_{p,i}\}, \tag{S63}$$

where $\mathscr{E}$ is the set of essential genes. Clearly, this assumption of independence fails in the context of polycistronic messages. We will discuss the significance of this feature of bacterial cells elsewhere, but we will ignore it in the current context. As we will discuss, the probability of arrest of any protein i to be above threshold is extremely small. It is therefore convenient to work in terms of the CDFs, which are very close to zero:

$$\ln P_+ \approx -\sum_{i \in \mathscr{E}} \gamma^-\left(\tfrac{\mu_{m,i}}{\ln 2}, \tfrac{n_{p,i}}{\varepsilon_i \ln 2}\right), \tag{S64}$$

where γ^- is the regularized lower incomplete gamma function (Eq. S85) and represents the probability of arrest.

4. *Single-gene equation*

By summing the fitness losses from the metabolic load and cell arrest (Eqs. S59, S62, and S64), we can write an expression for the growth rate including contributions from essential gene i:

$$\ln \tfrac{k}{k_0} = -\tfrac{\lambda+\varepsilon_i}{N_0}\mu_{m,i} - \tfrac{1}{\ln 2}\gamma^-\left(\tfrac{\mu_{m,i}}{\ln 2}, \tfrac{n_{p,i}}{\varepsilon \ln 2}\right), \tag{S65}$$

where the first term on the RHS represents the fitness loss due to the metabolic load and the second term represents the fitness loss due to stochastic cell arrest due to protein i falling below threshold.

5. *Optimization of transcription for bacteria*

The growth rate is:

$$\ln \tfrac{k}{k_0} = -(\Lambda + \tfrac{\varepsilon}{N_0})\mu_m - \tfrac{1}{\ln 2}\gamma^-\left(\tfrac{\mu_m}{\ln 2}, \tfrac{n_p}{\varepsilon \ln 2}\right), \tag{S66}$$

where γ^- is the regularized lower incomplete gamma function (Eq. S85), which is the CDF of the gamma distribution and represents the probability of arrest due to gene i. For bacteria, we consider the special case of optimizing the message number only at fixed translation efficiency [11, 53]. To determine the optimal transcription level, we set the partial derivative of Eq. S66 with respect to μ_m to zero. The optimum message number $\hat{\mu}_m$ satisfies the equation:

$$\tfrac{(\lambda+\varepsilon)\ln 2}{N_0} = -[\partial_{\hat{\mu}_m}\gamma(\hat{\mu}_m, \hat{n}_m)]_{\hat{n}_m = \frac{\hat{\mu}_m}{\hat{o}}}. \tag{S67}$$

We define the relative load:

$$\Lambda \equiv \tfrac{(\lambda+\varepsilon)}{N_0}, \tag{S68}$$

and substitute this into the optimum message number equation:

$$\Lambda \ln 2 = -[\partial_{\hat{\mu}_m}\gamma(\hat{\mu}_m, \hat{n}_m)]_{\hat{n}_m = \frac{\hat{\mu}_m}{\hat{o}}}, \tag{S69}$$

which is solved numerically.

6. *Estimate of the relative load in bacterial cells*

In bacterial cells, we will assume a constant translation efficiency model [11]. We therefore use the modified relative load formula (Eq. S68) to estimate Λ. We will assume that the load is dominated by proteins and messages:

$$N_0 = \sum_i (\lambda + \varepsilon)\mu_{m,i} = (\lambda + \varepsilon)N_m, \tag{S70}$$

where N_m is the total number of messages. We can then solve this equation for Λ:

$$\hat{\Lambda} = \tfrac{\lambda+\varepsilon}{N_0} = \tfrac{1}{N_m} \approx 10^{-5}, \tag{S71}$$

based on the total message number estimate for *E. coli* [11].

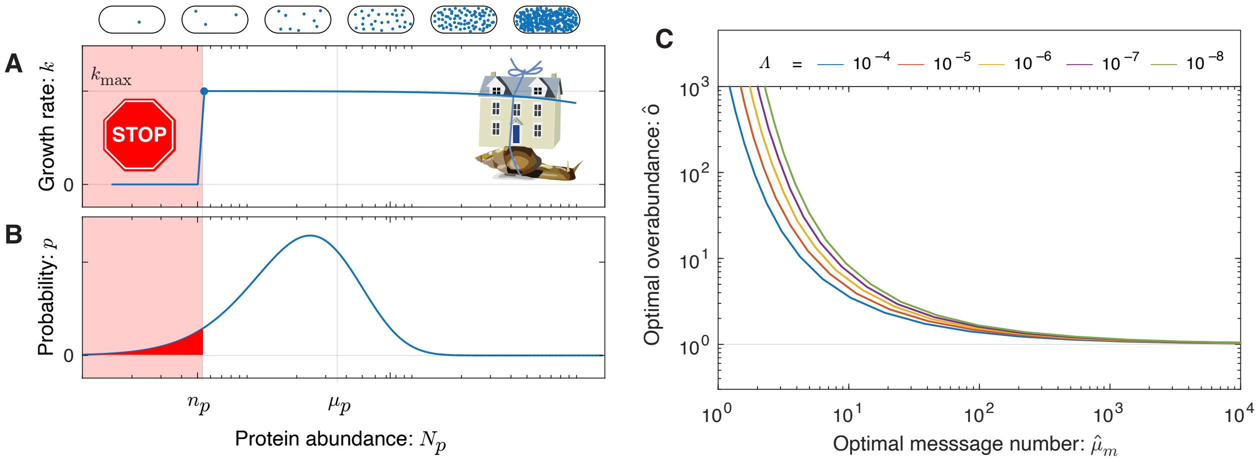

FIG. S14. **The RLTO model predicts that protein overabundance is fitness optimizing. Panel A: The fitness landscape is asymmetric in the RLTO model.** Motivated by single-cell growth data, cell fitness is modeled using the Robustness-Load Trade-Off model (RLTO). In the model, there is a metabolic cost of protein expression which favors low expression; however, growth arrests for protein number N_p smaller than the threshold level n_p (red). The relative metabolic cost of overabundance is small relative to the cost of growth arrest due to the large number of proteins synthesized, resulting in a highly asymmetric fitness landscape [11]. **Panel B: The gene expression process is stochastic.** There is significant cell-to-cell variation in protein abundance (N_p) around the mean level (μ_p). Even for mean expression levels significantly above the threshold level n_p, some cells fall below threshold (red). The distribution in protein number is modeled using a gamma distribution [53]. **Panel C: Overabundance is predicted to optimize cell fitness.** The asymmetry of the fitness landscape drives the optimal protein expression level to be overabundant ($\mu_p > n_p$). The RLTO model makes a quantitative prediction of the optimal overabundance ($\hat{o} \equiv \hat{\mu}_p/n_p$) as a function of the message number μ_m and a global parameter, the relative load $\Lambda \approx 10^{-5}$ (red curve). Overabundance is predicted to be extremely high ($o \gg 1$) for low expression genes ($\mu_m \approx 1$) and much closer to sufficiency ($o \approx 1$) for high expression genes ($\mu_m \gg 10$). Although the optimal overabundance depends on the relative load Λ, its qualitative dependence is unchanged over orders of magnitude in variation of the parameter.

C. Results: The fitness landscape of the RLTO model is highly asymmetric

In the RLTO model, the fitness landscape for a single cell is determined by an asymmetric fitness landscape: Protein underabundance is extremely costly due to the risk of growth arrest, while the cost of protein overabundance is only associated with an increase in metabolic load. (See Fig. S14A.) Naïvely, this tradeoff predicts that the cell maximizes its fitness by simply expressing just above the minimum protein threshold for function [7]. However, achieving growth robustness at a population level is nontrivial. Gene expression is stochastic [39], leading to significant cell-to-cell variation in protein numbers, which we model with a gamma distribution (Fig. S14B) [35, 36]. Therefore, the strong asymmetry of the fitness landscape predicts protein overabundance [11].

D. Results: The RLTO model predicts overabundance is optimal for low-expression proteins

The optimal regulatory program for transcription and translation (μ_m and ε values) can be predicted analytically. The values are determined by a single global parameter, the relative load Λ, and the gene-specific threshold number n_p. The threshold number is not directly observable experimentally; instead we predict the optimal overabundance o, defined as the ratio of the mean protein number to the threshold number:

$$o \equiv \mu_p/n_p. \tag{S72}$$

As shown in [11], by taking partial derivatives of the relative growth rate (Eq. S66) with respect to message number and translation efficiency, respectively, we can define the optimal overabundance:

$$\hat{o} \equiv \frac{\hat{\mu}_p}{n_p} = \frac{\hat{\varepsilon}\hat{\mu}_m}{n_p}, \tag{S73}$$

in the large multiplicity limit where the overall metabolic load is much smaller than the metabolic load for a single gene: $N_0 \gg (\lambda + \hat{\varepsilon})\hat{\mu}_m$. The optimal overabundance can be rewritten to find the optimization condition for message number:

$$\Lambda \ln 2 \;=\; -\partial_{\hat{\mu}_m}\gamma(\tfrac{\hat{\mu}_m}{\ln 2}, \tfrac{\hat{\mu}_m}{\hat{o}\ln 2}). \tag{S74}$$

As seen in Fig. S14C, the RLTO model generically predicts that for a range of relative loads, the optimal protein fraction is overabundant ($o > 1$); however, overabundance is not uniform for all proteins, but rather

depends on transcription. For highly-transcribed genes ($\mu_m \gg 1$), the overabundance is predicted to be quite small ($o \approx 1$); however, for lowly-transcribed genes (message numbers approaching unity), the overabundance is predicted to be extremely high ($o \gg 1$).

6. METHODS: STATISTICAL PROCEDURES

In this section, we provide a summary of statistical approaches that are common to the analyses in the paper.

A. Maximum Likelihood Estimation

The maximum likelihood (*i.e.* minimum information) estimates (MLE) of the parameters are defined:

$$\hat{\theta}^i = \arg\min_{\theta^i} h(\mathrm{data}|\theta^i). \tag{S75}$$

In all instances, these optimizations are performed numerically, either by direct minimization of the Shannon information (h), or for normal models, by least-squares minimization.

B. Parametric uncertainty estimates

To estimate the parameter uncertainty in the analysis of datasets, we use the Cramer-Rao bound to estimate of the uncertainty from the Fisher information [46]:

$$\sigma_{\theta^i} = \sqrt{[\hat{I}^{-1}]^{ii}}, \tag{S76}$$

where σ_{θ^i} is the estimate of the standard error for parameter θ^i, $\hat{I}$ is the estimator of the Fisher information, and $[\hat{I}^{-1}]^{ii}$ is the ii component of the inverse Fisher information matrix. For each statistical model, we describe how the Fisher information is estimated in detail (Hessian or Jacobian *etc*).

C. Null-hypothesis-testing approach

For null-hypothesis testing, we define two sequential null-hypothesis tests of nested statistical models. If the initial null hypothesis is rejected, we then interpret the initial alternative hypothesis as the updated null hypothesis and adopt the remaining model as the alternative hypothesis. For each test, we will use a Likelihood Ratio Test (LRT) where we define the test statistic λ in terms of the Shannon information:

$$\lambda = h_0 - h_1, \tag{S77}$$

where h_0 and h_1 are the Shannon information for the null and alternative hypotheses respectively. We will assume the Wilks' theorem: *I.e.* the test statistic Λ under the null hypothesis will have a chi-squared distribution [61, 62]:

$$\Lambda \sim \tfrac{1}{2}\chi^2_{\Delta K}, \tag{S78}$$

where the degrees-of-freedom $\Delta K = 1$ is equal to the difference between the dimension of the alternative and null models. (The factor of 1/2 appears in this equation, since the test statistic is defined by the Shannon information difference rather than the deviance [46].) The p-value can then be computed:

$$p = \Pr\{\Lambda > \lambda\} = \gamma^+(\tfrac{1}{2}\Delta K, \lambda), \tag{S79}$$

where γ^+ is the upper regularized incomplete gamma function (Eq. S95), $\Delta K = 1$ is the difference in model dimensions, and λ is the test statistic [46].

7. DISTRIBUTIONS AND CONVENTIONS

A. Gamma distribution conventions

There are a number of conflicting conventions for the gamma function and distribution arguments. We will use those defined on Wikipedia and the CRC Encyclopedia of Mathematics [63].

The gamma distributed random variable X will be written:

$$X \sim \Gamma(a, \theta), \tag{S80}$$

where a is the shape parameter and θ is the scale parameter. The PDF of the distribution is:

$$p_X(x|a,\theta) \equiv \tfrac{x^{a-1}}{\theta^a \Gamma(a)} e^{-x/\theta}, \tag{S81}$$

where $\Gamma(a)$ is the gamma function. The CDF is therefore:

$$\begin{aligned} P_X(x|a,\theta) &\equiv \Pr\{X < x|a,\theta\}, & \text{(S82)} \\ &= \int_0^x \mathrm{d}x'\, p_\Gamma(x'|a,\theta), & \text{(S83)} \\ &= \int_0^{x/\theta} \mathrm{d}x''\, \tfrac{x''^{a-1}}{\Gamma(a)} e^{-x}, & \text{(S84)} \\ &= \gamma^-(a, x/\theta), & \text{(S85)} \end{aligned}$$

where γ^- is the regularized lower incomplete gamma function. The survival function is:

$$\begin{aligned} \Pr\{X > x|a,\theta\} &= 1 - P_X(x|a,\theta), & \text{(S86)} \\ &= \int_{x/\theta}^{\infty} \mathrm{d}x''\, \tfrac{x''^{a-1}}{\Gamma(a)} e^{-x}, & \text{(S87)} \\ &= \gamma^+(a, x/\theta), & \text{(S88)} \end{aligned}$$

where γ^+ is the regularized upper incomplete gamma function.

B. Chi-squared distribution conventions

In statistical null hypothesis testing, the chi-squared distribution arises in the context of the Likelihood Ratio Test (LRT). Let Y be distributed like a chi-squared with k degrees of freedom:

$$Y \sim \chi^2_k, \tag{S89}$$

where the PDF is:

$$p_Y(y|k) = \frac{1}{2^{k/2}\Gamma(k/2)} y^{k/2-1} e^{-y/2}, \tag{S90}$$

where Γ is the gamma function. The CDF is therefore:

$$\begin{aligned} P_Y(y|k) &\equiv \Pr\{Y < y|k\}, && \text{(S91)}\\ &= \int_0^y \mathrm{d}y'\ p_Y(y'|k), && \text{(S92)}\\ &= \int_0^x \mathrm{d}x'\ p_X(x'|\tfrac{k}{2}, 2), && \text{(S93)}\\ &= \gamma^-(\tfrac{k}{2}, \tfrac{y}{2}), && \text{(S94)} \end{aligned}$$

where p_X is the PDF of the gamma distribution (Eq. S81) and γ^- is the regularized lower incomplete gamma function. The survival function is:

$$\Pr\{Y > y|k\} = \gamma^+(\tfrac{k}{2}, \tfrac{y}{2}), \tag{S95}$$

where γ^+ is the regularized upper incomplete gamma function.

8. DESCRIPTION OF SUPPLEMENTARY DATA

A. Data Tables

Data S1: Overabundance for all genes as measured by TFNseq analysis. The original TFNseq data was previously generated by the Manoil lab [18]. Format: Open Document Format (xlsx).

Data S2: A list of essential genes ranked by overabundance. Format: Open Document Format (xlsx).

Data S3: Representative single-cell imaging-based cell cytometry data for wild-type *A. baylyi* proliferating on minimal media (Km$^-$) from a single progenitor cell (Sec. 1 D). Format: Open Document Format (xlsx).

Data S4: Representative single-cell imaging-based cell cytometry data for *A. baylyi* Δ*IS* proliferating on minimal media (Km$^+$) from a single progenitor cell in a knockout-depletion experiment (Sec. 1 D). Format: Open Document Format (xlsx).

Data S5: Representative single-cell imaging-based cell cytometry data for *A. baylyi* Δ*dnaA* proliferating on minimal media (Km$^+$) from a single progenitor cell in a knockout-depletion experiment (Sec. 1 E). Format: Open Document Format (xlsx).

Data S6: Representative single-cell imaging-based cell cytometry data for *A. baylyi* Δ*dnaN* proliferating on minimal media (Km$^+$) from a single progenitor cell in a knockout-depletion experiment (Sec. 1 F). Format: Open Document Format (xlsx).

Data S7: Representative single-cell imaging-based cell cytometry data for *A. baylyi* Δ*murA* proliferating on minimal media (Km$^+$) from a single progenitor cell in a knockout-depletion experiment (Sec. 1 H). Format: Open Document Format (xlsx).

Data S8: Representative single-cell imaging-based cell cytometry data for *A. baylyi* Δ*ftsN* proliferating on minimal media (Km$^+$) from a single progenitor cell in a knockout-depletion experiment (Sec. 1 G). Format: Open Document Format (xlsx).

B. Annotated sequences

Data S9: The annotated sequence of the DnaN fluorescent fusion *YPet-dnaN*. Format: Genbank file.

C. Supplemental movies

Movie S1: Wild-type *A. baylyi* proliferating on minimal media (Km$^-$). Frame rate: 1 frame/2 min. (Sec. 1 D.) Raw images.

Movie S2: Wild-type *A. baylyi* proliferating on minimal media (Km$^-$). Frame rate: 1 frame/2 min. (Sec. 1 D.) Annotated/segmented images.

Movie S3: *A. baylyi* Δ*IS* proliferating on minimal media (Km$^+$) in a knockout-depletion experiment. Frame rate: 1 frame/3 min. (Sec. 1 D.) Raw images.

Movie S4: *A. baylyi* Δ*IS* proliferating on minimal media (Km$^+$) in a knockout-depletion experiment. Frame rate: 1 frame/3 min. (Sec. 1 D.) Annotated/segmented images.

Movie S5: *A. baylyi* Δ*dnaA* proliferating on minimal media (Km$^+$) in a knockout-depletion experiment. Frame rate: 1 frame/2 min. (Sec. 1 E.) Raw images.

Movie S6: *A. baylyi* Δ*dnaA* proliferating on minimal media (Km$^+$) in a knockout-depletion experiment. Frame rate: 1 frame/2 min. (Sec. 1 E.) Annotated/segmented images.

Movie S7: *A. baylyi* Δ*dnaN* proliferating on minimal media (Km$^+$) in a knockout-depletion experiment. Frame rate: 1 frame/9 min. (Sec. 1 F.) Raw images.

Movie S8: *A. baylyi* Δ*dnaN* proliferating on minimal media (Km$^+$) in a knockout-depletion experiment. Frame rate: 1 frame/9 min. (Sec. 1 F.) Annotated/segmented images.

Movie S9: *A. baylyi* Δ*murA* proliferating on minimal media (Km^+) in a knockout-depletion experiment. Frame rate: 1 frame/2 min. (Sec. 1 H.) Raw images.

Movie S10: *A. baylyi* Δ*murA* proliferating on minimal media (Km^+) in a knockout-depletion experiment. Frame rate: 1 frame/2 min. (Sec. 1 H.) Annotated/segmented images.

Movie S11: *A. baylyi* Δ*ftsN* proliferating on minimal media (Km^+) in a knockout-depletion experiment. Frame rate: 1 frame/2 min. (Sec. 1 G.) Raw images.

Movie S12: *A. baylyi* Δ*ftsN* proliferating on minimal media (Km^+) in a knockout-depletion experiment. Frame rate: 1 frame/2 min. (Sec. 1 G.) Annotated/segmented images.

REFERENCES

1. B. Alberts, A. Johnson, J. Lewis, M. Raff, K. Roberts, P. Walter, *Molecular Biology of the Cell* (Garland, ed. 4, 2002).

2. E. Dekel, U. Alon, Optimality and evolutionary tuning of the expression level of a protein. *Nature* **436**, 588–592 (2005).

3. J.-B. Lalanne, D. J. Parker, G.-W. Li, Spurious regulatory connections dictate the expression-fitness landscape of translation factors. *Mol. Syst. Biol.* **17**, e10302 (2021).

4. J. W. Lengeler, G. Drews, H. G. Schlegel, Eds., *Biology of the Prokaryotes* (Georg Thieme Verlag, 1998); 10.1002/9781444313314.

5. M. Kafri, E. Metzl-Raz, F. Jonas, N. Barkai, Rethinking cell growth models. *FEMS Yeast Res.* **16**, fow081 (2016).

6. J. Hausser, A. Mayo, L. Keren, U. Alon, Central dogma rates and the trade-off between precision and economy in gene expression. *Nat. Commun.* **10**, 68 (2019).

7. N. M. Belliveau, G. Chure, C. L. Hueschen, H. G. Garcia, J. Kondev, D. S. Fisher, J. A. Theriot, R. Phillips, Fundamental limits on the rate of bacterial growth and their influence on proteomic composition. *Cell Syst.* **12**, 924–944.e2 (2021).

8. J. M. Peters, A. Colavin, H. Shi, T. L. Czarny, M. H. Larson, S. Wong, J. S. Hawkins, C. H. S. Lu, B. M. Koo, E. Marta, A. L. Shiver, E. H. Whitehead, J. S. Weissman, E. D. Brown, L. S. Qi, K. C. Huang, C. A. Gross, A comprehensive, CRISPR-based functional analysis of essential genes in bacteria. *Cell* **165**, 1493–1506 (2016).

9. S. Donati, M. Kuntz, V. Pahl, N. Farke, D. Beuter, T. Glatter, J. V. Gomes-Filho, L. Randau, C. Y. Wang, H. Link, Multi-omics analysis of CRISPRi-knockdowns identifies mechanisms that buffer decreases of enzymes in E. coli metabolism. *Cell Syst.* **12**, 56–67.e6 (2021).

10. T. Baba, H.-C. Huan, K. Datsenko, B. L. Wanner, H. Mori, The applications of systematic in-frame, single-gene knockout mutant collection of *Escherichia coli* K-12. *Methods Mol. Biol.* **416**, 183–194 (2008).

11. T. W. Lo, H. J. Choi, D. Huang, P. A. Wiggins, Noise robustness and metabolic load determine the principles of central dogma regulation. *Sci. Adv.* **10**, eado3095 (2024).

12. K. E. McGinness, T. A. Baker, R. T. Sauer, Engineering controllable protein degradation. *Mol. Cell* **22**, 701–707 (2006).

13. J. H. Davis, T. A. Baker, R. T. Sauer, Small-molecule control of protein degradation using split adaptors. *ACS Chem. Biol.* **6**, 1205–1213 (2011).

14. D. E. Cameron, J. J. Collins, Tunable protein degradation in bacteria. *Nat. Biotechnol.* **32**, 1276–1281 (2014).

15. X. Liu, C. Gallay, M. Kjos, A. Domenech, J. Slager, Sebastiaan P van Kessel, K. Knoops, R. A. Sorg, J.-R. Zhang, J.-W. Veening, High-throughput CRISPRi phenotyping identifies new essential genes in *Streptococcus pneumoniae*. *Mol. Syst. Biol.* **13**, 931 (2017).

16. S. N. J. Franks, R. Heon-Roberts, B. J. Ryan, CRISPRi: A way to integrate iPSC-derived neuronal models. *Biochem. Soc. Trans.* **52**, 539–551 (2024).

17. J. Bailey, J. Cass, J. Gasper, N.-D. Ngo, P. Wiggins, C. Manoil, Essential gene deletions producing gigantic bacteria. *PLOS Genet.* **15**, e1008195 (2019).

18. L. A. Gallagher, J. Bailey, C. Manoil, Ranking essential bacterial processes by speed of mutant death. *Proc. Natl. Acad. Sci. U.S.A.* **117**, 18010–18017 (2020).

19. K. T. Elliott, E. L. Neidle, *Acinetobacter baylyi* ADP1: Transforming the choice of model organism. *IUBMB Life* **63**, 1075–1080 (2011).

20. D. Metzgar, J. M. Bacher, V. Pezo, J. Reader, V. Döring, P. Schimmel, P. Marlière, V. de Crécy-Lagard, *Acinetobacter sp.* ADP1: An ideal model organism for genetic analysis and genome engineering. *Nucleic Acids Res.* **32**, 5780–5790 (2004).

21. S. M. Mangiameli, C. N. Merrikh, P. A. Wiggins, H. Merrikh, Transcription leads to pervasive replisome instability in bacteria. *eLife* **6**, e19848 (2017).

22. S. M. Mangiameli, B. T. Veit, H. Merrikh, P. A. Wiggins, The replisomes remain spatially proximal throughout the cell cycle in bacteria. *PLOS Genet.* **13**, e1006582 (2017).

23. S. M. Mangiameli, J. A. Cass, H. Merrikh, P. A. Wiggins, The bacterial replisome has factory-like localization. *Curr. Genet.* **64**, 1029–1036 (2018).

24. N. J. Kuwada, B. Traxler, P. A. Wiggins, Genome-scale quantitative characterization of bacterial protein localization dynamics throughout the cell cycle. *Mol. Microbiol.* **95**, 64–79 (2015).

25. G. M. Cooper, *The Cell: A Molecular Approach* (Sinauer Associates 2000, ed. 2, 2000).

26. K. P. Lemon, A. D. Grossman, Localization of bacterial dna polymerase: Evidence for a factory model of replication. *Science* **282**, 1516–1519 (1998).

27. R. Reyes-Lamothe, D. J. Sherratt, M. C. Leake, Stoichiometry and architecture of active DNA replication machinery in *Escherichia coli*. *Science* **328**, 498–501 (2010).

28. K. J. Cutler, C. Stringer, T. W. Lo, L. Rappez, N. Stroustrup, S. Brook Peterson, P. A. Wiggins, J. D. Mougous, Omnipose: A high-precision morphology-independent solution for bacterial cell segmentation. *Nat. Methods* **19**, 1438–1448 (2022).

29. V. de Berardinis, D. Vallenet, V. Castelli, M. Besnard, A. Pinet, C. Cruaud, S. Samair, C. Lechaplais, G. Gyapay, C. Richez, M. Durot, A. Kreimeyer, F. L. Fèvre, V. Schächter, V. Pezo, V. Döring, C. Scarpelli, C. Médigue, G. N. Cohen, P. Marlière, M. Salanoubat, J. Weissenbach, A complete collection of single-gene deletion mutants of *Acinetobacter baylyi* ADP1. *Mol. Syst. Biol.* **4**, 174 (2008).

30. S. Autret, A. Levine, I. B. Holland, S. J. Séror, Cell cycle checkpoints in bacteria. *Biochimie* **79**, 549–554 (1997).

31. J. S. Hawkins, M. R. Silvis, B. M. Koo, J. M. Peters, H. Osadnik, M. Jost, C. C. Hearne, J. S. Weissman, H. Todor, C. A. Gross, Mismatch-CRISPRi reveals the co-varying expression-fitness relationships of essential genes in *Escherichia coli* and *Bacillus subtilis*. *Cell Syst.* **11**, 523–535.e9 (2020).

32. L. Keren, J. Hausser, M. Lotan-Pompan, I. Vainberg Slutskin, H. Alisar, S. Kaminski, A. Weinberger, U. Alon, R. Milo, E. Segal, Massively parallel interrogation of the effects of gene expression levels on fitness. *Cell* **166**, 1282–1294.e18 (2016).

33. B. Bosch, M. DeJesus, N. C. Poulton, W. Zhang, C. A. Engelhart, A. Zaveri, S. Lavalette, N. Ruecker, C. Trujillo, J. B. Wallach, S. Li, S. Ehrt, B. T. Chait, D. Schnappinger, J. M. Rock, Genome-wide gene expression tuning reveals diverse vulnerabilities of *M. tuberculosis*. *Cell* **184**, 4579–4592.e24 (2021).

34. D. L. Nelson, M. M. Cox, *Lehninger Principles of Biochemistry* (W.H. Freeman, ed. 7, 2017).

35. J. Paulsson, M. Ehrenberg, Random signal fluctuations can reduce random fluctuations in regulated components of chemical regulatory networks. *Phys. Rev. Lett.* **84**, 5447–5450 (2000).

36. N. Friedman, L. Cai, X. S. Xie, Linking stochastic dynamics to population distribution: An analytical framework of gene expression. *Phys. Rev. Lett.* **97**, 168302 (2006).

37. G. Lambert, E. Kussell, Memory and fitness optimization of bacteria under fluctuating environments. *PLOS Genet.* **10**, e1004556 (2014).

38. M. Mori, S. Schink, D. W. Erickson, U. Gerland, T. Hwa, Quantifying the benefit of a proteome reserve in fluctuating environments. *Nat. Commun.* **8**, 1225 (2017).

39. J. M. Raser, E. K. O'Shea, Noise in gene expression: Origins, consequences, and control. *Science* **309**, 2010–2013 (2005).

40. D. Weichart, N. Querfurth, M. Dreger, R. Hengge-Aronis, Global role for ClpP-containing proteases in stationary-phase adaptation of *Escherichia coli*. *J. Bacteriol.* **185**, 115–125 (2003).

41. A. L. Goldberg, A. C. St John, Intracellular protein degradation in mammalian and bacterial cells: Part 2. *Annu. Rev. Biochem.* **45**, 747–803 (1976).

42. M. Gupta, A. N. T. Johnson, E. R. Cruz, E. J. Costa, R. L. Guest, S. H.-J. Li, E. M. Hart, T. Nguyen, M. Stadlmeier, B. P. Bratton, T. J. Silhavy, N. S. Wingreen, Z. Gitai, M. Wühr, Global protein turnover quantification in *Escherichia coli* reveals cytoplasmic recycling under nitrogen limitation. *Nat. Commun.* **15**, 5890 (2024).

43. V. Barbe, D. Vallenet, N. Fonknechten, A. Kreimeyer, S. Oztas, L. Labarre, S. Cruveiller, C. Robert, S. Duprat, P. Wincker, L. N. Ornston, J. Weissenbach, P. Marlière, G. N. Cohen, C. Médigue, Unique features revealed by the genome sequence of *Acinetobacter* sp. ADP1, a versatile and naturally transformation competent bacterium. *Nucleic Acids Res.* **32**, 5766–5779 (2004).

44. J. Miller, *Experiments in Molecular Genetics* (Cold Spring Harbor Laboratory, 1972).

45. A. C. Chang, S. N. Cohen, Construction and characterization of amplifiable multicopy DNA cloning vehicles derived from the P15A cryptic miniplasmid. *J. Bacteriol.* **134**, 1141–1156 (1978).

46. D. R. Cox, D. V. Hinkley, *Theoretical Statistics* (Chapman & Hall, 1974).

47. T. W. Lo, K. J. Cutler, S. Stylianidou, C. Brennan, S. B. Nissen, N. J. Kuwada, P. A. Wiggins, OmniSegger, GitHub (2024); https://github.com/tlo-bot/omnisegger.

48. S. Stylianidou, C. Brennan, S. B. Nissen, N. J. Kuwada, P. A. Wiggins, SuperSegger: Robust image segmentation, analysis and lineage tracking of bacterial cells. *Mol. Microbiol.* **102**, 690–700 (2016).

49. T. W. Lo, K. J. Cutler, H. J. Choi, P. A. Wiggins, Omnisegger: A time-lapse image analysis pipeline for bacterial cells. bioRxiv 625259 [Preprint] (2024). https://doi.org/10.1101/2024.11.25.625259.

50. K. P. Burnham, D. R. Anderson, Multimodel inference: Understanding AIC and BIC in model selection. *Social. Methods Res.* **33**, 261–304 (2004).

51. P. D. Karp, S. Paley, R. Caspi, A. Kothari, M. Krummenacker, P. E. Midford, L. R. Moore, P. Subhraveti, S. Gama-Castro, V. H. Tierrafria, P. Lara, L. Muñiz-Rascado, C. Bonavides-Martinez, A. Santos-Zavaleta, A. Mackie, G. Sun, T. A. Ahn-Horst, H. Choi, M. W. Covert, J. Collado-Vides, I. Paulsen, The EcoCyc database (2023). *EcoSal Plus* **11**, eesp-0002-2023 (2023).

52. P. H. Culviner, C. K. Guegler, M. T. Laub, A simple, cost-effective, and robust method for rRNA depletion in RNA-sequencing studies. *mBio* **11**, e00010–e00020 (2020).

53. Y. Taniguchi, P. J. Choi, G. W. Li, H. Chen, M. Babu, J. Hearn, A. Emili, X. S. Xie, Quantifying *E. coli* proteome and transcriptome with single-molecule sensitivity in single cells. *Science* **329**, 533–538 (2010).

54. F. Crick, Central dogma of molecular biology. *Nature* **227**, 561–563 (1970).

55. J. L. Hargrove, F. H. Schmidt, The role of mRNA and protein stability in gene expression. *FASEB J.* **3**, 2360–2370 (1989).

56. A. L. Koch, H. R. Levy, Protein turnover in growing cultures of *Escherichia coli*. *J. Biol. Chem.* **217**, 947–957 (1955).

57. M. Martin-Perez, J. Villén, Determinants and regulation of protein turnover in yeast. *Cell Syst.* **5**, 283–294.e5 (2017).

58. M. Scott, C. W. Gunderson, E. M. Mateescu, Z. Zhang, T. Hwa, Interdependence of cell growth and gene expression: Origins and consequences. *Science* **330**, 1099–1102 (2010).

59. E. Levien, J. Min, J. Kondev, A. Amir, Non-genetic variability in microbial populations: Survival strategy or nuisance? *Rep. Prog. Phys.* **84**, 116601 (2021).

60. E. O. Powell, Growth rate and generation time of bacteria, with special reference to continuous culture. *Microbiology* **15**, 492–511 (1956).

61. S. S. Wilks, The large-sample distribution of the likelihood ratio for testing composite hypotheses. *Ann. Math. Stat.* **9**, 60–62 (1938).

62. G. Casella, R. Berger, *Statistical Inference* (Duxbury Resource Center, 2001).

63. E. W. Weisstein, *CRC Encyclopedia of Mathematics* (Chapman & Hall/CRC, 2009).
